# Ddc2^ATRIP^ promotes Mec1^ATR^ activation at RPA-ssDNA tracts

**DOI:** 10.1371/journal.pgen.1008294

**Published:** 2019-08-01

**Authors:** Himadri Biswas, Greicy Goto, Weibin Wang, Patrick Sung, Katsunori Sugimoto

**Affiliations:** 1 Department of Microbiology, Biochemistry and Molecular Genetics, International Center for Public Health, New Jersey Medical School, Rutgers, The State University of New Jersey, Newark, New Jersey, United States of America; 2 Department of Molecular Biophysics and Biochemistry, Yale University School of Medicine, New Haven, Connecticut, United States of America; 3 Department of Biochemistry and Structural Biology, University of Texas Health Science Center at San Antonio, San Antonio, Texas, United States of America; Columbia University, UNITED STATES

## Abstract

The DNA damage checkpoint response is controlled by the phosphatidylinositol 3-kinase-related kinases (PIKK), including ataxia telangiectasia-mutated (ATM) and ATM and Rad3-related (ATR). ATR forms a complex with its partner ATRIP. In budding yeast, ATR and ATRIP correspond to Mec1 and Ddc2, respectively. ATRIP/Ddc2 interacts with replication protein A-bound single-stranded DNA (RPA-ssDNA) and recruits ATR/Mec1 to sites of DNA damage. Mec1 is stimulated by the canonical activators including Ddc1, Dpb11 and Dna2. We have characterized the *ddc2-S4* mutation and shown that Ddc2 not only recruits Mec1 to sites of DNA damage but also stimulates Mec1 kinase activity. However, the underlying mechanism of Ddc2-dependent Mec1 activation remains to be elucidated. Here we show that Ddc2 promotes Mec1 activation independently of Ddc1/Dpb11/Dna2 function *in vivo* and through ssDNA recognition *in vitro*. The *ddc2-S4* mutation diminishes damage-induced phosphorylation of the checkpoint mediators, Rad9 and Mrc1. Rad9 controls checkpoint throughout the cell-cycle whereas Mrc1 is specifically required for the S-phase checkpoint. Notably, S-phase checkpoint signaling is more defective in *ddc2-S4* mutants than in cells where the Mec1 activators (Ddc1/Dpb11 and Dna2) are dysfunctional. To understand a role of Ddc2 in Mec1 activation, we reconstituted an *in vitro* assay using purified Mec1-Ddc2 complex, RPA and ssDNA. Whereas ssDNA stimulates kinase activity of the Mec1-Ddc2 complex, RPA does not. However, RPA can promote ssDNA-dependent Mec1 activation. Neither ssDNA nor RPA-ssDNA efficiently stimulates the Mec1-Ddc2 complex containing Ddc2-S4 mutant. Together, our data support a model in which Ddc2 promotes Mec1 activation at RPA-ssDNA tracts.

## Author summary

The ATR-ATRIP complex is recruited to sites of DNA damage by replication protein A-bound single-stranded DNA (RPA-ssDNA), and controls cellular responses to various types of DNA damage. The recruitment of ATR-ATRIP to RPA-ssDNA is not sufficient for the kinase activation. The activation of ATR-ATRIP requires activators including TopBP1 and ETAA1. In budding yeast, Mec1-Ddc2 (the ATR-ATRIP homolog) is activated through a similar mechanism. Activation of Mec1-Ddc2 requires Ddc1 (Rad9 homolog), Dpb11 (TopBP1 homolog) and Dna2. Our studies of the *ddc2-S4* mutation have shown that Ddc2 promotes not only Mec1 recruitment but also Mec1 activation. In this study we show that Ddc2 promotes Mec1 activation independently of Ddc1/Dpb11/Dna2 function *in vivo*. We also show that Ddc2 regulates Mec1 activation through ssDNA recognition *in vitro*. Although RPA does not directly stimulate Mec1 activity, it can enhance ssDNA-dependent Mec1 activation. Our results suggest that ATR/Mec1, like ATM/Tel1 and DNA-PKcs, is activated upon DNA damage recognition.

## Introduction

Chromosomes are constantly damaged by exogenous and endogenous threats [1]. The repair of damaged chromosomes is therefore critical for maintaining genome stability. The DNA damage response consists of multiple pathways controlled by the phosphatidylinositol 3-kinase-related kinases (PIKK) [2, 3]. These kinases include DNA-dependent protein kinase catalytic subunit (DNA-PKcs), ataxia telangiectasia-mutated (ATM), and ATM and Rad3-related (ATR). Although all these PIKKs respond to DNA damage, their DNA damage specificities are different. ATM and DNA-PKcs are activated by double-stranded DNA breaks (DSBs), whereas ATR responds to various types of DNA lesions with single-stranded DNA (ssDNA) [4, 5].

The Mre11-Rad50-Nbs1 complex recruits and activates ATM at DNA ends [6] whereas the Ku complex recruits and activates DNA-PKcs at DNA ends [3, 7]. The recruitment of ATM and DNA-PKcs is thus coupled to the kinase activation. Replication protein A (RPA) is the major protein that binds ssDNA with a high affinity [8]. ATR interacts with a partner ATRIP and recognizes RPA-covered ssDNA (RPA-ssDNA) [4, 5]. However, the recruitment of the ATR-ATRIP complex (ATR-ATRIP) to RPA-ssDNA is not sufficient for ATR activation. Indeed, ATR-ATRIP is stimulated by checkpoint regulators including TopBP1 and ETAA1 [4, 5]. TopBP1 is recruited to sites of DNA damage or stalled replication forks although the mechanism for the recruitment is not fully understood [9–12]. TopBP1 appears to engage with the Rad9-Rad1-Hus1 (9-1-1) complex at dsDNA-ssDNA junctions [4, 5]. Subsequently, TopBP1 directly stimulates the ATR-ATRIP kinase [4, 5, 13]. ETAA1 interacts with RPA and acts at stalled replication forks [14–16]. Like TopBP1, ETAA1 directly activates ATR-ATRIP [14, 15]. Thus, ATR-ATRIP is recruited by recognizing RPA-ssDNA and subsequently activated through multiple steps [4, 5].

In budding yeast, the Mec1-Ddc2 complex (Mec1-Ddc2) corresponds to ATR-ATRIP [17, 18]. Mec1-Ddc2 interacts with RPA-ssDNA to accumulate at sites of DNA damage [17]. The Ddc1-Mec3-Rad17 complex (the yeast 9-1-1 complex) recruits Dpb11 (TopBP1 ortholog) to the dsDNA-ssDNA junction [19, 20]. In budding yeast, both Ddc1^Rad9^ and Dpb11^TopBP1^ stimulate Mec1-Ddc2 kinase activity [21–25]. These observations have established the model in which the Ddc1-Dpb11 axis governs the checkpoint pathway by directly activating Mec1-Ddc2. In parallel with the Ddc1-Dpb11 axis, the Dna2 nuclease/helicase stimulates Mec1 kinase activity and controls DNA damage and replication checkpoints in S phase [26]. All Ddc1, Dpb11 and Dna2 proteins utilize the unstructured domains with aromatic amino acid residues (Trp or Tyr) to increase the catalytic activity of Mec1 [23, 24, 26, 27]. Thus, Ddc1, Dpb11 and Dna2 appear to activate Mec1 through a similar mechanism.

In budding yeast, Mec1 activates the downstream kinase Rad53 (Chk2 homolog) to enforce DNA damage checkpoint response [28, 29]. Signal transduction from Mec1 to Rad53 requires checkpoint mediators, such as Rad9 and Mrc1; Mec1 phosphorylates Rad9 and Mrc1 to promote their interaction with Rad53 at sites of DNA damage [30–33]. Rad9 controls checkpoint throughout the cell-cycle whereas Mrc1 is specifically required for the S phase DNA damage checkpoint [28, 32, 34–36]. Mrc1 associates with components of the replication fork in S phase [37, 38]. In contrast, recruitment of Rad9 to sites of DNA damage is a highly regulated process that involves three distinct mechanisms [39, 40]. First, the TUDOR domain of Rad9 interacts with K79-methylated histone H3 [41, 42]. Second, the tandem BRCT domain of Rad9 interacts with S129-phosphorylated histone H2A [43]. Finally, Rad9 binds to the Dpb11 scaffold protein [39, 44]. Histone H3 methylation is a constitutive mark on chromatin [45] and phosphorylated histone H2A spreads over around DNA lesions [46, 47]. However, the Dpb11 scaffold protein localizes to DNA lesions; indeed, Mec1 phosphorylates Ddc1 to promote Ddc1-Dpb11 interaction [39, 44, 48]. Thus, the Ddc1-Dpb11 axis not only stimulates Mec1 kinase activity but also promotes Rad9 recruitment to sites of DNA damage.

We have identified a separation-of-function *ddc2* mutation (*ddc2-S4*) that causes defects in Mec1 activation but does not affect Mec1 recruitment [49]. However, it is not known how Ddc2-dependent Mec1 activation triggers checkpoint signaling. Moreover, the underlying mechanism of Ddc2-dependent Mec1 activation remains to be determined. To understand the significance of Ddc2-dependent Mec1 activation, we further characterized the *ddc2-S4* mutation by carrying out genetic experiments. We found that the *ddc2-S4* mutation impaired Rad9 and Mrc1 phosphorylation after DNA damage, consistent with the previous observation that the *ddc2-S4* mutation is defective in checkpoint activation throughout the cell cycle [49]. The Ddc1-Dpb11 axis and Dna2 contribute to Mec1 activation in S phase [26]. We also found that S-phase checkpoint signaling is more significantly defective in *ddc2-S4* mutants than in *ddc1-* and *dna2*-deficient mutants. Thus, Ddc2 appears to promote Mec1 activation independently of Ddc1/Dpb11 and Dna2 function. We further examined the effect of *ddc2-S4* mutation on kinase activity of Mec1-Ddc2 using an *in vitro* reconstitution system. Whereas ssDNA stimulated Mec1 activity, RPA did not. However, RPA was found to promote ssDNA-dependent Mec1 activation. The Mec1-Ddc2 complex containing Ddc2-S4 mutant (Mec1-Ddc2-S4) exhibited a basal kinase activity *in vitro*. However, neither ssDNA nor RPA-ssDNA efficiently stimulated Mec1-Ddc2-S4. Our results support a model in which Ddc2 promotes Mec1 activation through ssDNA recognition.

## Results

### The *ddc2-S4* mutation impairs Rad9 phosphorylation and recruitment after DNA damage

**Fig 1:**
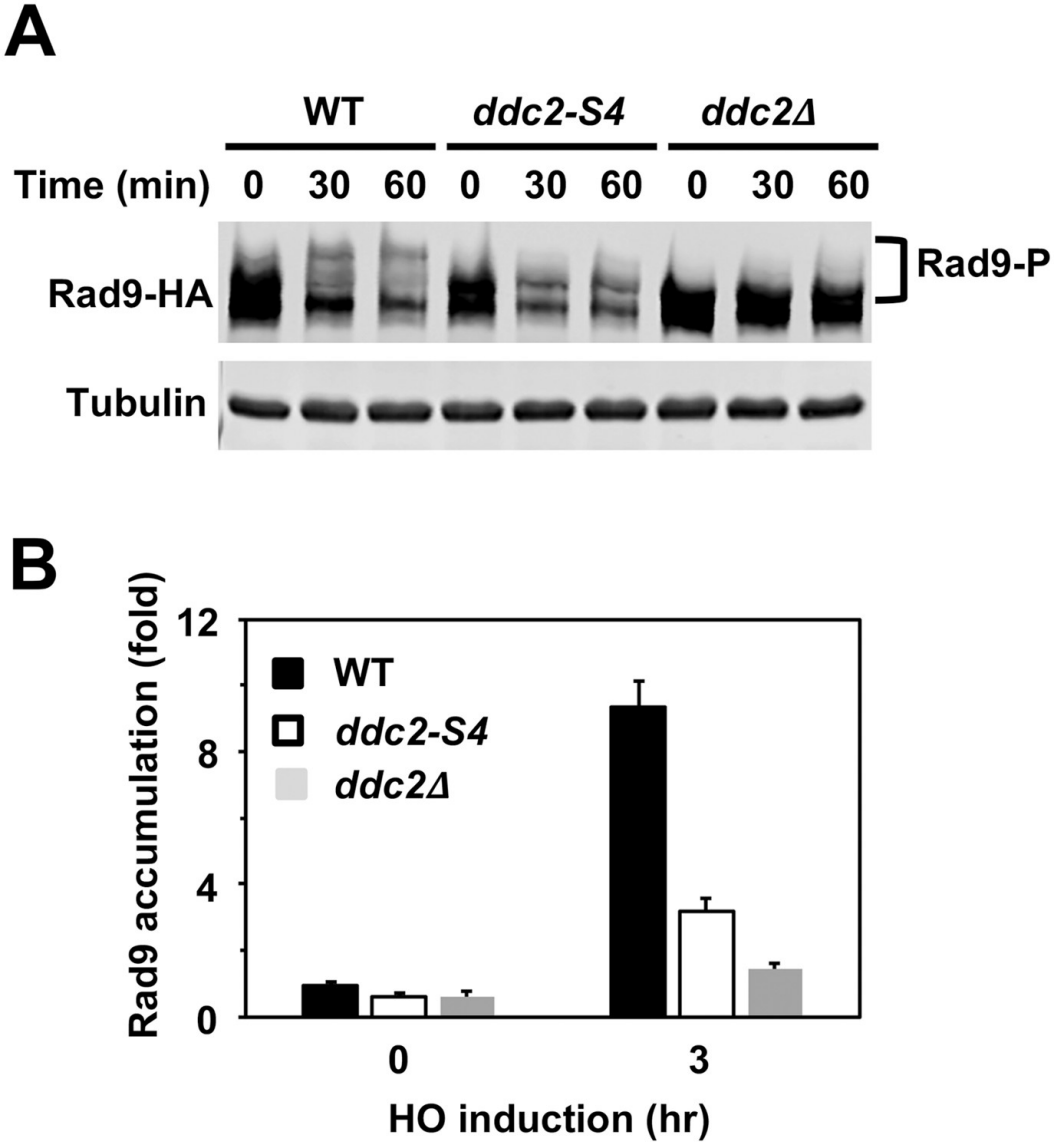
Effect of *ddc2-S4* or *ddc2*Δ mutation on Rad9 phosphorylation after DNA damage. (A) Wild-type (HB09), *ddc2-S4* (HB10) or *ddc2*Δ (KSC1536) cells expressing Rad9-HA were treated with nocodazole to arrest at G2/M. Cells were then exposed to 0.05% MMS. Cells were collected at the indicated time points, and extracts were subjected to immunoblotting analysis with anti-HA antibodies or anti-tubulin antibodies. (B) Effect of *ddc2-S4* or *ddc2*Δ on Rad9 localization to a HO-induced DSB. Wild-type (HB09), *ddc2-S4* (HB10) or *ddc2*Δ (KSC1536) cells expressing Rad9-HA were transformed with the YCpA-GAL-HO plasmid. Transformed cells were grown in sucrose and treated with nocodazole. After arrest at G2/M, the culture was incubated with galactose for 3 hr to induce HO expression, while half of the culture was maintained in sucrose to repress HO expression. Cells were subjected to chromatin immunoprecipitation with anti-HA antibodies. Association of Rad9 with a HO-induced DSB was analyzed by real-time PCR. Relative enrichment was determined from three independent experiments. The error bars indicate standard deviation.

Mec1 phosphorylates the Rad9 checkpoint mediator to promotes Rad9-Rad53 interaction, which is essential for Rad53 activation [30, 31]. The *ddc2-S4* mutation confers defects in DNA damage checkpoint activation and damage-induced Rad53 phosphorylation at G2/M [49]. To understand the significance of Ddc2-dependent Mec1 activation, we first examined the effect of *ddc2-S4* mutation on Rad9 phosphorylation after DNA damage at G2/M (Fig 1A). Cells expressing HA-tagged Rad9 protein (Rad9-HA) were arrested with nocodazole and exposed to methyl methanesulfonate (MMS). Cells were then subjected to immunoblotting analysis with anti-HA antibodies to monitor Rad9 phosphorylation (Fig 1A). Rad9 underwent phosphorylation in wild-type cells after MMS treatment. Phosphorylation was decreased in *ddc2-S4* mutants but less significantly compared with in *ddc2*Δ mutants. Thus, *the ddc2-S4* mutation impairs Rad9 phosphorylation after DNA damage.

One explanation for Rad9 phosphorylation defect in *ddc2-S4* mutants is that Rad9 does not efficiently localize to sites of DNA damage. We next examined the effect of *ddc2-S4* mutation on Rad9 accumulation at sites of DNA damage by chromatin immunoprecipitation (ChIP) assay (Fig 1B). In budding yeast, HO endonuclease introduces a sequence-specific DSB. We used an experimental system in which cells carry the GAL-HO plasmid and contain a single HO cleavage site at the *ADH4* locus [49]. In this system, HO endonuclease, expressed after incubation with galactose, generates a single DSB at the *ADH4* locus. Cells expressing HA-tagged Rad9 (Rad9-HA) protein were transformed with the GAL-HO plasmid. Transformants were grown initially in sucrose to repress HO expression, and then transferred to medium containing nocodazole to arrest at G2/M. After arrest, galactose was added to induce HO expression. Cells were then analyzed by the ChIP assay using anti-HA antibodies. Rad9 accumulated at sites of DNA damage less efficiently in *ddc2-S4* mutants than in wild-type cells. However, the *ddc2-S4* mutation conferred a milder defect in Rad9 accumulation compared with the *ddc2*Δ mutation (Fig 1B).

**Fig 2:**
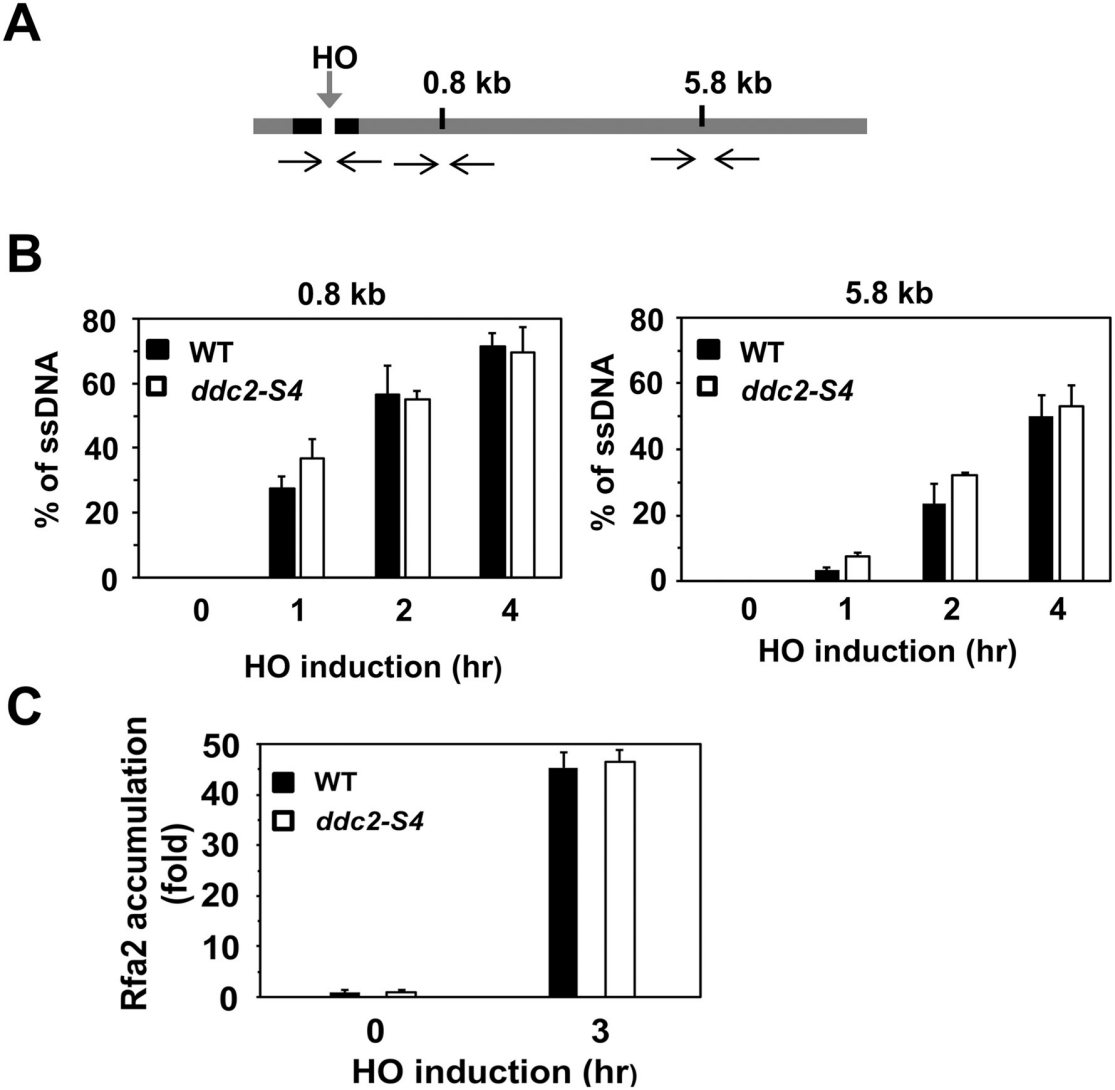
Effect of *ddc2-S4* mutation on DNA end resection and RPA accumulation. (A) Scheme of the *ADH4* locus containing a HO cleavage site. One EcoRI restriction site is located 0.8 kb away from the HO cleavage site whereas another is 5.8 kb away. The black arrows indicate PCR primer pairs to monitor HO or EcoRI cleavage. (B) Effect of *ddc2-S4* mutation on DNA end resection. Wild-type (HB01) or *ddc2-S4* (HB02) cells carrying YCpA-GAL-HO were grown in sucrose and treated with nocodazole. After arrest at G2/M, the culture was incubated with galactose to induce HO expression. Cells were collected at the indicated times for genomic DNA preparation. Genomic DNA was digested with EcoRI and analyzed by real-time PCR. Experiments were carried out three times. The error bars indicate standard deviation. (C) Effect of *ddc2-S4* on Rfa2 localization to a HO-induced DSB. Wild-type (HB01) or *ddc2-S4* (HB02) cells were transformed with the YCpA-GAL-HO plasmid. Transformed cells were grown in sucrose and treated with nocodazole. The culture was then incubated with galactose for 3 hr to induce HO expression, while half of the culture was maintained in sucrose to repress HO expression. Cells were subjected to chromatin immunoprecipitation with anti-Rfa2 antibodies. Association of Rfa2 with a HO-induced DSB was analyzed by real-time PCR. Relative enrichment was determined from three independent experiments. The error bars indicate standard deviation.

Rad9 limits the Sae2- and Sgs1-dependent pathway and interferes with DNA end resection [50]. We next addressed whether the *ddc2-S4* mutation affects the kinetics of DNA end resection. To this end we monitored ssDNA generation at two EcoRI restriction sites near the HO cleavage site (at 0.8 kb or 5.8 kb from the site) by a quantitative PCR-based method [51] (Fig 2A). PCR amplifies only resected DNA because the EcoRI restriction enzyme can cleave unprocessed DNA (S1 Fig). The PCR amplification, normalized to the efficiency of HO cleavage, corresponds to the rate of DNA end resection [51]. The *ddc2-S4* mutation did not significantly affect DNA end resection (Fig 2B). RPA, consisting of Rfa1, Rfa2 and Rfa3, binds to ssDNA tracts [8]. We also examined whether the *ddc2-S4* mutation affects RPA accumulation near the DSB (Fig 2C). No apparent defect in Rfa2 association was observed in *ddc2-S4* mutants. These result support the previous finding that the *ddc2-S4* mutation does not affect Mec1 localization to sites of DNA damage [49]. Our results are also consistent with the current view that Mec1 positively controls DNA end resection although it promotes Rad9 accumulation at sites of DNA damage [52].

**Fig 3:**
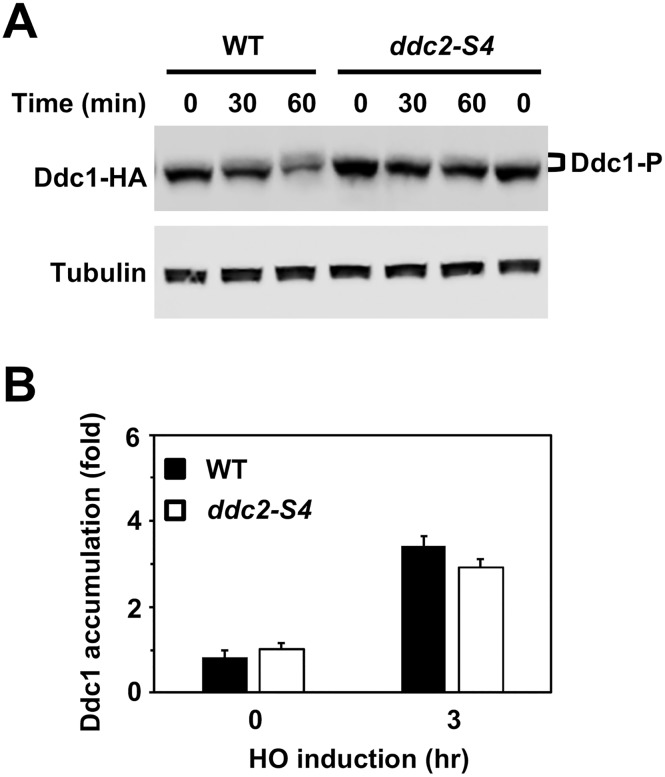
Effect of *ddc2-S4* on Ddc1 phosphorylation and localization in response to DNA damage. (A) Effect of *ddc2-S4* mutation on Ddc1 phosphorylation after DNA damage. Wild-type (HB12) or *ddc2-S4* (HB13) cells expressing Ddc1-HA were analyzed as in Fig 1A. (B) Effect of *ddc2-S4* mutation on Ddc1 localization to a HO-induced DSB. Wild-type (HB12) or *ddc2-S4* (HB13) cells expressing Ddc1-HA were transformed with the YCpA-GAL-HO plasmid. Transformed cells were cultured as in Fig 1B and subjected to ChIP assay to monitor Ddc1 localization. Relative enrichment was determined from three independent experiments. The error bars indicate standard deviation from three independent experiments.

Mec1 phosphorylates Ddc1 to recruit Rad9 near sites of DNA damage through the Dpb11 scaffold [39, 44, 48]. We next examined the effect of *ddc2-S4* mutation on Ddc1 phosphorylation after DNA damage (Fig 3A). Cells expressing HA-tagged Ddc1 protein were treated as above and subjected to immunoblotting analysis to monitor Ddc1 phosphorylation. Ddc1 phosphorylation was decreased in *ddc2-S4* mutants compared with wild-type cells. Mec1 and Ddc1 are independently recruited to sites of DNA damage [53, 54]. We confirmed that the *ddc2-S4* mutation has no impact on Ddc1 accumulation at sites of DNA damage (Fig 3B). Thus, the *ddc2-S4* mutation impairs Ddc1 phosphorylation that promotes Ddc1-Dpb11-Rad9 assembly at sites of DNA damage [39, 44, 48].

### The *ddc2-S4* mutation is defective in S phase checkpoint signaling

**Fig 4:**
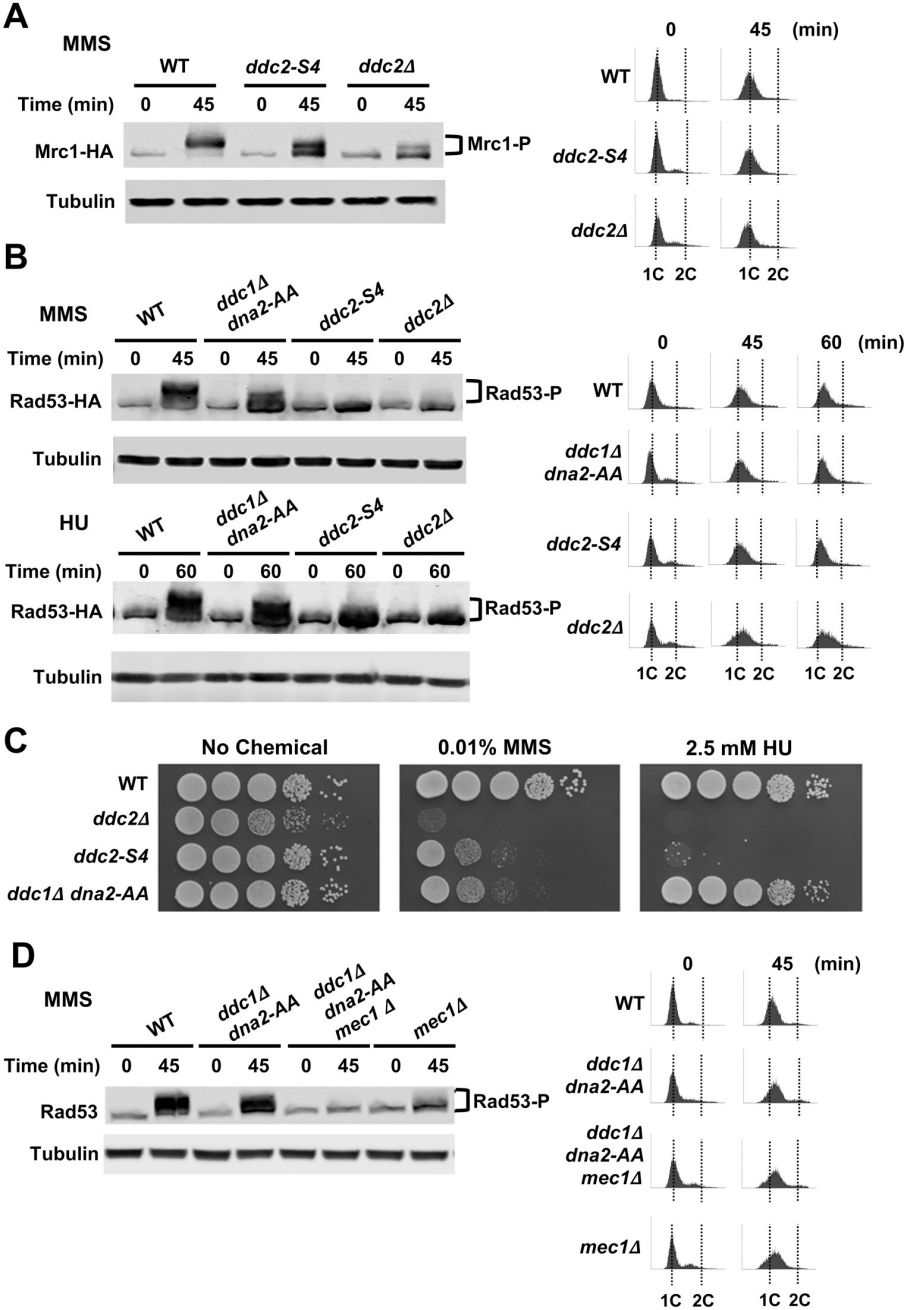
Effect of *ddc2-S4* mutation on S-phase checkpoint signaling. (A) Wild-type (KSC4233), *ddc2-S4* (KSC4234) and *ddc2*Δ (KSC4235) cells expressing Mrc1-HA were arrested with α-factor at G1 and released into medium containing 0.05% MMS. Cells were collected at the indicated time and analyzed as in Fig 1A. Cell cycle progression from G1 to S phase was monitored by DNA flow cytometry. (B) Wild-type (KSC1178), *ddc1*Δ *dna2-AA* (KSC4219), *ddc2-S4* (KSC3153) and *ddc2*Δ (KSC1234) cells carrying the YCpT-Rad53-HA plasmid were synchronized with α-factor at G1 and released into medium containing 0.05% MMS or 100 mM HU. Cells were collected at the indicated time (45 min for MMS and 60 min for HU) and analyzed as in Fig 4A. (C) Effect of *ddc1*Δ *dna2-AA* or *ddc2-S4* on sensitivities to MMS and HU. Wild-type (KSC1178), *ddc1*Δ *dna2-AA* (KSC4219), *ddc2-S4* (KSC3153) and *ddc2*Δ (KSC1234) cells were serially diluted and spotted on plates medium containing MMS or HU. (D) Wild-type (KSC1178), *ddc1*Δ *dna2-AA* (KSC4219), *ddc1*Δ *dna2-AA mec1*Δ (KSC4238) or *mec1*Δ (KSC1186) cells were synchronized with α-factor at G1 and released into medium containing 0.05% MMS. Cells were collected at the indicated time and subjected to immunoblotting analysis with anti-Rad53 or anti-tubulin antibody. Cell cycle progression from G1 to S phase was monitored by DNA flow cytometry.

Mec1 phosphorylates the Mrc1 checkpoint mediator that is essential for Rad53 activation during S phase [32, 35]. Notably, Mrc1-dependent Rad53 activation does not require Rad9 or Ddc1/Dpb11 function [55]. We further examined the effect of *ddc2-S4* mutation on Mrc1 phosphorylation after DNA damage (Fig 4A). Wild-type, *ddc2-S4* or *ddc2*Δ cells expressing HA-tagged Mrc1 protein were arrested in G1 with α-factor and released into medium containing MMS. Cells were harvested at the indicated times after release. We confirmed that cells remained within S phase at the time point after release (Fig 4A). Mrc1 phosphorylation was decreased in *ddc2-S4* mutants but less significantly than in *ddc2*Δ mutants. Thus, Ddc2-dependent Mec1 activation also controls the Mrc1-dependent checkpoint pathway during S phase.

The Ddc1-Dpb11 axis and Dna2 control DNA damage checkpoints in S phase [26]. Two substitution mutations at the N-terminus of Dna2 (*dna2-W128A*, *Y130A*; hereafter called *dna2-AA*) abrogate its checkpoint function [26]. We next compared Rad53 phosphorylation in *ddc2-S4*, *ddc2Δ* and *ddc1Δ dna2-AA* mutants in S phase (Fig 4B). Cells expressing HA-tagged Rad53 protein were arrested at G1 and released into MMS or hydroxyurea (HU) as above. Rad53 phosphorylation was decreased in *ddc1Δ dna2-AA* mutants. However, a more significant defect was observed in *ddc2-S4* or *ddc2Δ* mutants (Fig 4B). Our results agree with the previous observation that *ddc1Δ dna2-AA* cells are less defective in Rad53 activation than *mec1*Δ cells [56]. We next compared DNA damage sensitivity of *ddc2-S4* and *ddc1Δ dna2-AA* mutants. While the *ddc2-S4* and the *ddc1Δ dna2-AA* mutation caused similar sensitivities to MMS, *ddc2-S4* mutants were more sensitive to HU than *ddc1Δ dna2-AA* mutants (Fig 4C). We further addressed whether the residual checkpoint activation in *ddc1Δ dna2-AA* mutants depends on Mec1 function in S phase (Fig 4D). The introduction of a *mec1*Δ mutation decreased damage-induced Rad53 phosphorylation in *ddc1Δ dna2-AA* mutants. Together, these results suggest that Ddc2 promotes Mec1 activation through a Ddc1/Dpb11/Dna2-independent mechanism.

### The *ddc2-S4* mutation is defective in RPA phosphorylation *in vivo* and *in vitro*

**Fig 5:**
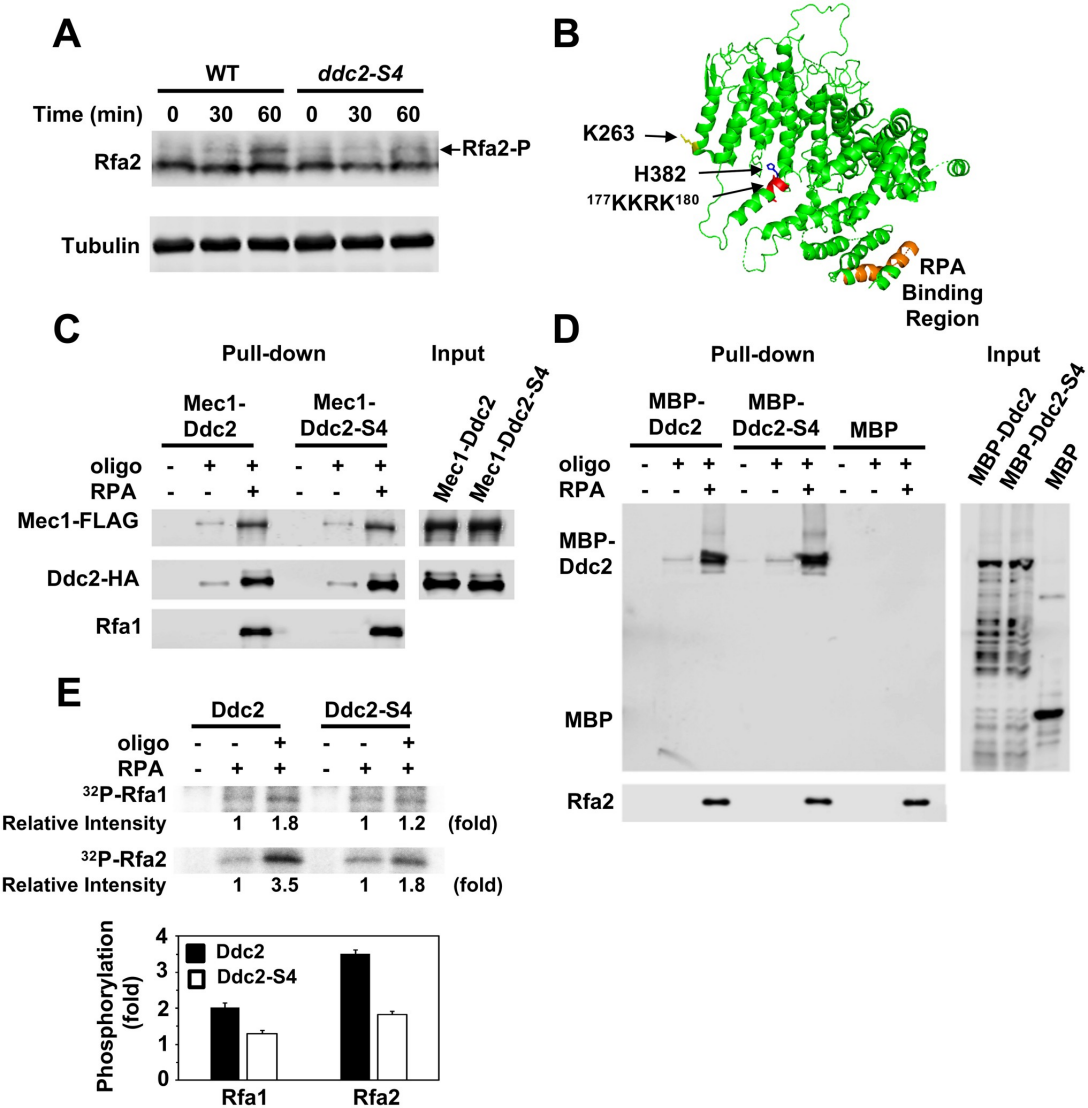
Effect of *ddc2-S4* mutation on RPA phosphorylation *in vivo* and *in vitro*. (A) Rfa2 phosphorylation after exposure to MMS. Wild-type (HB01) or *ddc2-S4* (HB02) cells were cultured as in Fig 1A and subjected to immunoblotting analysis with anti-Rfa2 antibodies. (B) Position of the *ddc2-S4* substitution mutation sites. The putative DNA binding (^177^KKRK^180^) and the RPA binding (amino acid 10 to 30) [64] region are highlighted in red and orange, respectively. The side chain of K263 and H382 residues is shown in yellow and blue, respectively. (C) Effect of *ddc2-S4* mutation on ssDNA-binding of Mec1-Ddc2. Streptavidin beads were first incubated with RPA (1 pmol) or bio-oligo(dN)_80_ (5 pmol). Beads were further incubated with Mec1-Ddc2 or Mec1-Ddc2-S4 (0.5 pmol). Captured proteins on beads were detected by immunoblotting with anti-FLAG, anti-HA or anti-Rfa1 antibodies. Note that Mec1 is FLAG-tagged and Ddc2 is HA-tagged. (D) Effect of *ddc2-S4* mutation on ssDNA-binding of Ddc2. Streptavidin beads were first incubated with RPA (1 pmol) or bio-oligo(dN)_80_ (5 pmol). Beads were further incubated with MBP, MBP-Ddc2 or MBP-Ddc2-S4 (0.5 pmol). MBP or MBP-fusion proteins were prepared from *E*. *coli*. Captured proteins on beads were analyzed by immunoblotting with anti-MBP or anti-Rfa2 antibodies. (E) Effect of *ddc2-S4* mutation on RPA phosphorylation *in vitro*. Kinase reactions were carried out with Mec1-Ddc2 or Mec1-Ddc2-S4 (5 nM) in the absence or the presence of RPA (10 nM) or bio-oligo(dN)_80_ (125 nM). Incorporation of ^32^P into Rfa1 and Rfa2 were normalized to that observed with Rfa1 and Rfa2 alone. The error bars indicate standard deviation from three independent experiments.

Mec1 phosphorylates two subunits of RPA, Rfa1 and Rfa2, in response to DNA damage [57, 58] although the significance of RPA phosphorylation in checkpoint signaling is not fully understood [59]. We examined the effect of *ddc2-S4* mutation on Rfa2 phosphorylation after DNA damage (Fig 5A). Wild-type and *ddc2-S4* mutants were arrested with nocodazole at G2/M and exposed to MMS. Cells were then analyzed by immunoblotting with anti-Rfa2 antibodies. We found that damage-induced Rfa2 phosphorylation was decreased in *ddc2-S4* mutants. The *ddc2-S4* mutation does not affect Mec1 or RPA accumulation at sites of DNA damage [49] (Fig 2C). Thus, the *ddc2-S4* mutation impairs Rfa2 phosphorylation *in vivo*.

Mec1/ATR phosphorylates RPA efficiently in the presence of ssDNA *in vitro* [21, 58, 60]. However, whether RPA or ssDNA modulates ATR/Mec1 activity remains to be determined. To understand a role of Ddc2 in Mec1 activation, we reconstituted an *in vitro* system using purified Mec1-Ddc2 and RPA proteins. We have purified Mec1-Ddc2 and Mec1-Ddc2-S4 through a two-step affinity chromatography after overexpressing FLAG-tagged Mec1 and His-tagged Ddc2 protein in yeast cells (S2 and S3 Figs). In agreement with the previous studies [21, 58], Mec1-Ddc2 phosphorylated RPA efficiently in the presence of ssDNA (S4 Fig).

The *ddc2-S4* mutation contains two substation mutations (K263E, H382Y). K263 is implicated in Mec1-Ddc2 homodimerization [61] whereas H382 is in close proximity to the ^177^KKRK^180^ motif which is involved in DNA binding [61, 62] (Fig 5B, S5 Fig). Ddc2/ATRIP itself interacts weakly with ssDNA but RPA stimulates ssDNA binding of Ddc2/ATRIP [17, 62]. We determined the effect of *ddc2-S4* mutation on the interaction of Mec1-Ddc2 with ssDNA or RPA-ssDNA by a pull-down assay. Mec1-Ddc2 and Mec1-Ddc2-S4 were found to bind similarly to ssDNA or RPA-ssDNA *in vitro* (Fig 5C), consistent with the observation that the *ddc2-S4* mutation does not affect Mec1 accumulation at sites of DNA damage [49]. We further examined the effect of *ddc2-S4* mutation on Ddc2-ssDNA binding. We prepared MBP-fused Ddc2 or Ddc2-S4 from *E*. *coli* and further examined whether they interact with oligonucleotides in the presence or absence of RPA (Fig 5D). MBP-Ddc2 and MBP-Ddc2-S4 interacted similarly with ssDNA and RPA-ssDNA. MBP alone did not exhibit oligonucleotide binding (Fig 5D). Thus, the *ddc2-S4* mutation does not affect its own ssDNA- or RPA-ssDNA-binding abilities.

We next examined whether Mec1-Ddc2-S4, like Mec1-Ddc2, phosphorylates RPA efficiently in the presence of ssDNA (Fig 5E). Mec1-Ddc2 and Mec1-Ddc2-S4 similarly phosphorylated Rfa2 in the absence of ssDNA. However, in the presence of ssDNA, Mec1-Ddc2-S4 phosphorylated Rfa2 less efficiently compared with Mec1-Ddc2 (Fig 5E). A similar defect in Rfa1 phosphorylation was observed with Mec1-Ddc2-S4 (Fig 5E). Together, these results raise a possibility that Ddc2 upregulates Mec1 kinase activity by interacting with ssDNA or RPA-ssDNA.

### ssDNA, but not RPA, stimulates Mec1 activity in a Ddc2-dependent manner

**Fig 6:**
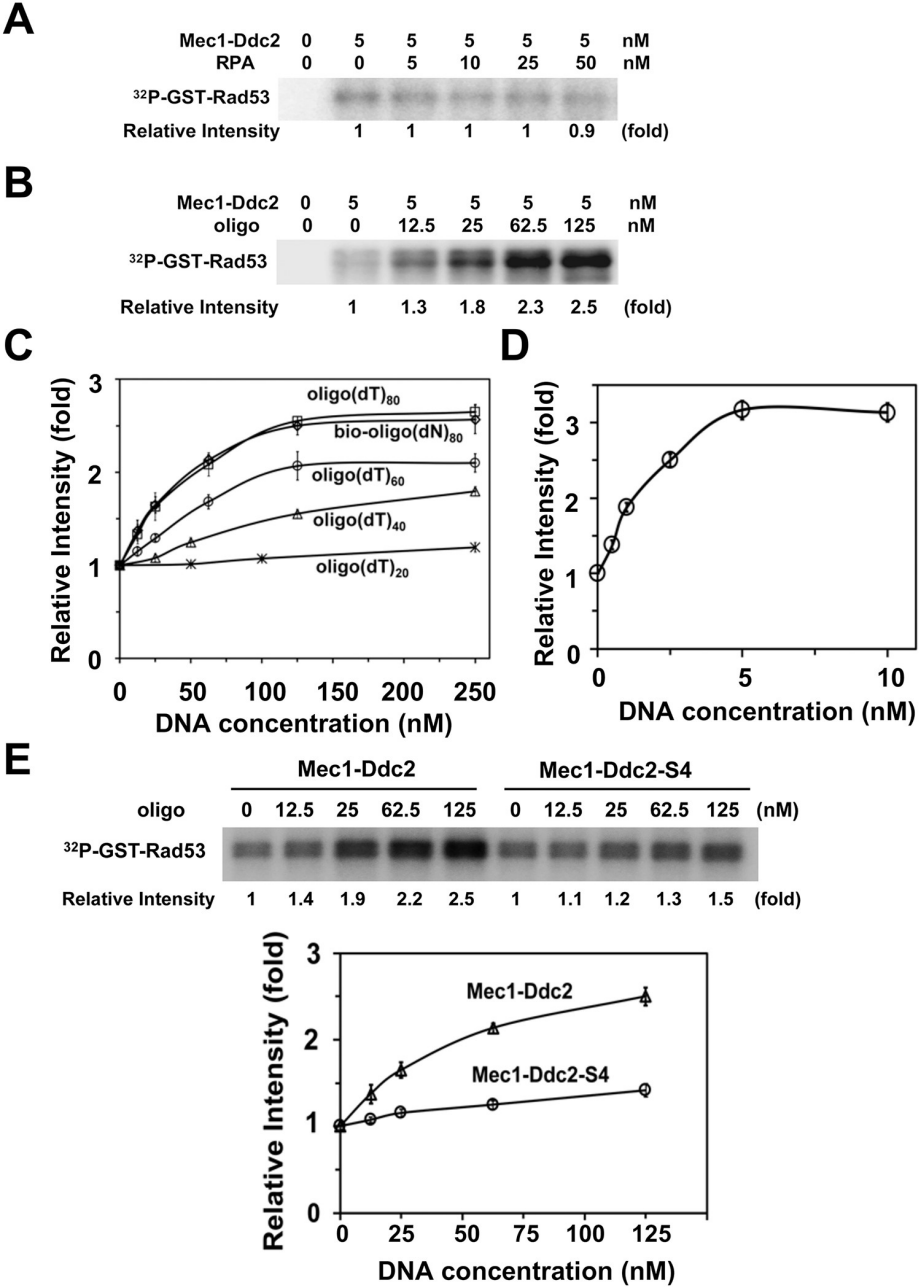
Effect of RPA or ssDNA addition on Mec1 activity. (A) Effect of RPA addition on Mec1 catalytic activity. Kinase reactions were carried out with or without Mec1-Ddc2 (5 nM) using various concentrations of RPA. GST-Rad53 C-terminus fusion (GST-Rad53) was used as a substrate of Mec1. Incorporation of ^32^P into GST-Rad53 was detected by phosphor imaging. The Rad53 C-terminus does not contain its kinase domain; phosphorylation of GST-Rad53 depends on Mec1-Ddc2. Phosphorylation levels of GST-Rad53 were normalized to that observed with Mec1-Ddc2 alone. The error bars indicate standard deviation from three independent experiments. (B) Effect of ssDNA addition on Mec1 catalytic activity. Kinase reactions were carried out with or without Mec1-Ddc2 (5 nM) using various concentrations of 80-mer biotin-oligo(dN)_80_. Incorporation of ^32^P into GST-Rad53 was analyzed as in A. The error bars indicate standard deviation from three independent experiments. (C, D) Effect of length and base-composition of ssDNA on Mec1 activation. Mec1-Ddc2 complex (5 nM) was incubated with various concentrations of oligonucleotides (20, 40, 60, 80-mer oligo(dT), 80-mer bio-oligo(dN)_80_ (C) or ΦX phage ssDNA (5 kb) (D). Phosphorylation of GST-Rad53 was normalized to that observed without RPA or ssDNA. The error bars indicate standard deviation from three independent experiments. (E) Effect of *ddc2-S4* mutation on Mec1 activation by ssDNA. Kinase reactions were carried out with Mec1-Ddc2 or Mec1-Ddc2-S4 (5 nM) using various concentrations of bio-oligo(dN)_80_. Incorporation of ^32^P into GST-Rad53 was analyzed as in A. The error bars indicate standard deviation from three independent experiments.

We addressed whether RPA or ssDNA regulates Mec1 activity using GST-Rad53 as a substrate (Fig 6). GST-Rad53 lacks the N-terminal kinase domain of Rad53; therefore, no phospho-incorporation into GST-Rad53 was observed without Mec1-Ddc2 [49] (Fig 6A and 6B). We first tested the effect of various concentrations of RPA on Mec1 kinase activity. RPA had no significant impact on Mec1 activity using GST-Rad53 as a substrate (Fig 6A).

We next investigated the effect of ssDNA on Mec1 activity using various lengths (20, 40, 60 or 80 mer) of oligo(dT) (Fig 6B and 6C). Although no apparent stimulation was observed with a 20 mer oligo(dT) (oligo(dT)_20_), longer oligonucleotides, oligo(dT)_40_, oligo(dT)_60_ and oligo(dT)_80_, were found to increase Mec1 activity more efficiently. Similar activation was observed with oligo(dT)_80_ and an 80-mer biotinylated oligonucleotide containing all DNA bases (bio-oligo(dN)_80_) (Fig 6C). We note that biotinylation of oligonucleotide has no impact on Mec1 activation (S6 Fig). High concentrations of 80-mer oligonucleotides (125 nM) were required to reach maximum activation compared with the concentration of Mec1 (5 nM). We further tested whether longer ssDNA stimulates Mec1 more strongly using 5 kb ΦX174 phage ssDNA (Fig 6D). A single-stranded form of ΦX174 phage stimulated Mec1 at much lower concentrations compared with 80-mer oligonucleotides. The maximum activation obtained with ΦX (3 fold) was slightly higher than that of 80-mer oligonucleotides (2.5 fold) (Fig 6C and 6D). These results indicate that ssDNA stimulates Mec1 in a dosage-dependent and length-dependent manner.

We then determined the effect of *ddc2-S4* mutation on ssDNA-dependent Mec1 activation using 80-mer oligonucleotides (Fig 6E). Mec1-Ddc2-S4 and Mec1-Ddc2 exhibited similar basal kinase activities. However, Mec1-Ddc2-S4 was not efficiently stimulated by ssDNA. Thus, ssDNA appears to stimulate Mec1 activity through a Ddc2-dependent mechanism.

### RPA modulates ssDNA-dependent Mec1 activation *in vitro*

**Fig 7:**
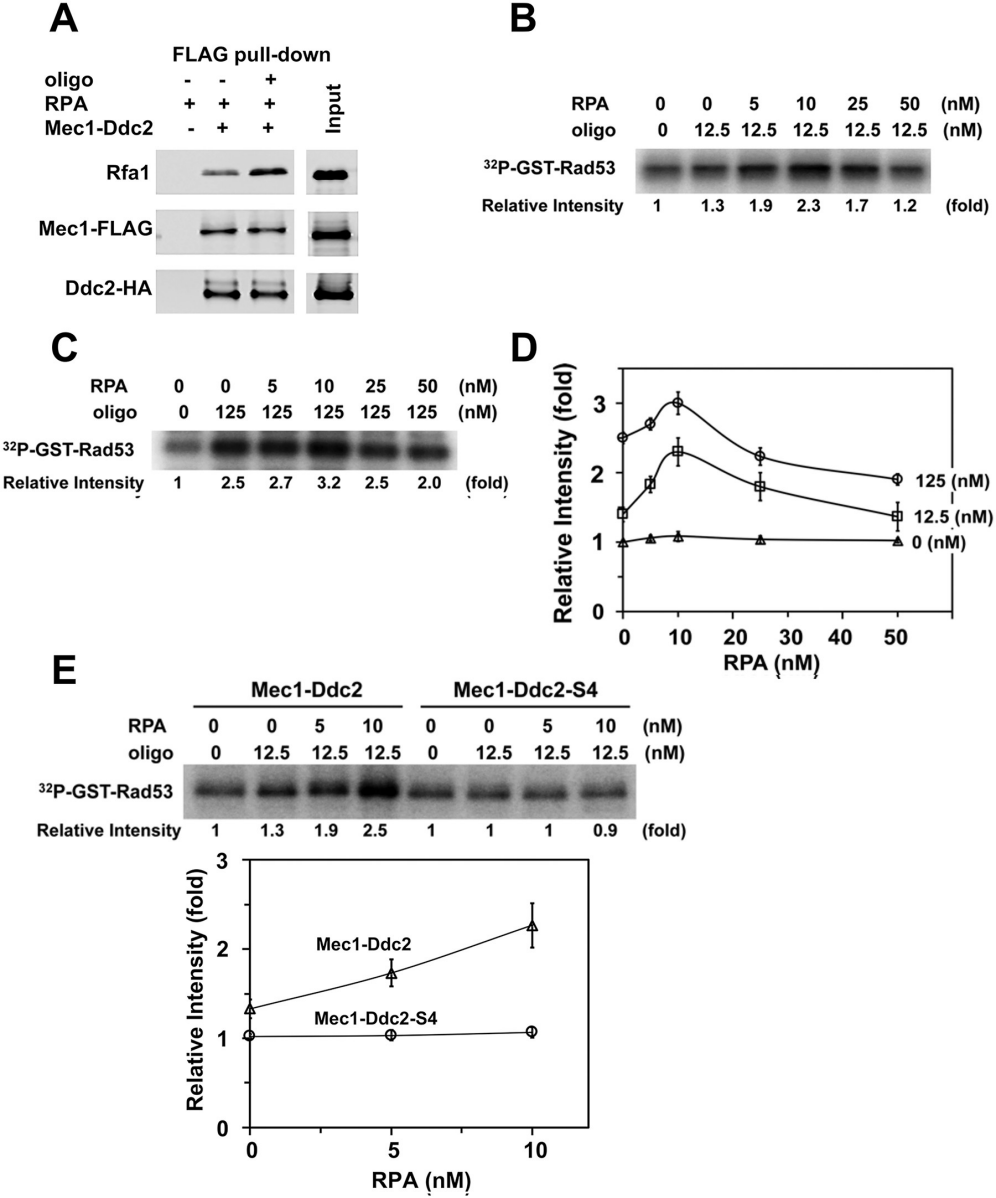
Effects of RPA on ssDNA-dependent Mec1 activation. (A) Interaction of Mec1-Ddc2 with RPA in the presence or absence of ssDNA. Mec1-Ddc2 (0.5 pmol) and RPA (1 pmol) were incubated with ANTI-FLAG-M2 agarose in the presence or absence of bio-oligo(dN)_80_ (5 pmol). Proteins bound to ANTI-FLAG-M2 agarose were analyzed by immunoblotting analysis with anti-FLAG, anti-HA or anti-Rfa1 antibodies. Note that Mec1 is FLAG-tagged and Ddc2 is HA-tagged. (B) Effect of RPA addition on Mec1 activation in the presence of low concentrations of ssDNA. Kinase reactions were carried out with Mec1-Ddc2 (5 nM) using various concentrations of RPA in the absence or presence of bio-oligo(dN)_80_ (12.5 nM). Incorporation of ^32^P into GST-Rad53 was analyzed as in Fig 6A. Kinase activities of Mec1-Ddc2, normalized to that observed with Mec1-Ddc2 alone, are shown in comparison with those in the presence of RPA or ssDNA. Experiments were carried out three times and the representative result is shown. (C) Effect of RPA on Mec1 activity in the presence of high concentrations of ssDNA. Kinase reactions were carried out with Mec1-Ddc2 (5 nM) using various concentrations of RPA in the absence or presence of bio-oligo(dN)_80_ (125 nM). Incorporation of ^32^P into GST-Rad53 was analyzed as in Fig 6A. Kinase activities of Mec1-Ddc2, normalized to that observed with Mec1-Ddc2 alone, are shown in comparison with those in the presence of RPA or ssDNA. Experiments were carried out three times and the representative result is shown. (D) Effects of RPA concentrations on ssDNA-dependent Mec1 activation. Kinase activities of Mec1-Ddc2 using various concentrations of RPA in the presence of bio-oligo(dN)_80_ (12.5 nM or 125 nM) were normalized to that observed with Mec1-Ddc2 alone (See Fig 7B or 7C, respectively). Relative kinase activities with various concentrations of RPA in the absence of ssDNA are also included (see Fig 6A). The error bars indicate standard deviation from three independent experiments. (E) Effect of *ddc2-S4* mutation on Mec1 activation by RPA and ssDNA. Kinase reactions were carried out with Mec1-Ddc2 or Mec1-Ddc2-S4 (5 nM) using various concentrations of RPA in the absence or the presence of bio-oligo(dN)_80_ (12.5 nM). Incorporation of ^32^P into GST-Rad53 was analyzed as in Fig 6A. The error bars indicate standard deviation from three independent experiments.

We next determined the combination effect of RPA and ssDNA on Mec1 kinase activity using GST-Rad53 as a substrate. RPA prompts ssDNA binding of Mec1-Ddc2 or Ddc2 (Fig 5C and 5D) whereas ssDNA stimulates Mec1 kinase activity (Fig 6). We thus expected that RPA promotes ssDNA-dependent Mec1 activation. However, Mec1-Ddc2 was found to interact with RPA independently of ssDNA *in vitro*, in agreement with the current view that the N-terminus of Rfa1 interacts directly with the N-terminus of Ddc2 [63, 64] (Fig 7A). Therefore, RPA by itself could compete with ssDNA-bound RPA for Mec1-Ddc2 binding (S7 Fig). Moreover, RPA is a good substrate of Mec1 (S4 Fig); that is, RPA could compete with GST-Rad53 as a Mec1 substrate [65]. Hence, high RPA concentrations could have negative impacts on ssDNA-dependent Mec1 activation *in vitro*.

We first examined the effect of RPA on Mec1 activation with a low concentration of 80-mer oligonucleotides (12.5 nM) (Fig 7B). We note that only weak Mec1 activation was observed at this concentration (Fig 6B and 6C). We incubated oligonucleotides with various concentrations of RPA and subsequently with Mec1-Ddc2 to initiate the kinase reaction. Lower concentrations of RPA enhanced Mec1 kinase activity whereas higher concentrations of RPA attenuated (Fig 7B and 7D). We further tested the effect of RPA on Mec1 activation with a higher concentration of oligonucleotide (125 nM) (Fig 7C). Again, lower concentrations of RPA stimulated Mec1 activity whereas higher concentrations of RPA attenuated. These results are consistent with the hypothesis that RPA promotes ssDNA-dependent Mec1 activation although high RPA concentrations have negative impacts on ssDNA-dependent Mec1 activation *in vitro*. The stimulatory effect of RPA was less pronounced when Mec1-Ddc2 was incubated with a higher concentration of oligonucleotides (Fig 7C and 7D), consistent with the observation that ssDNA, but not RPA, stimulates Mec1-Ddc2 activity (Fig 6).

As discussed above, the *ddc2-S4* mutation causes a defect in ssDNA-dependent Mec1 activation (Fig 6E) although it does not affect RPA-ssDNA binding of Mec1-Ddc2 (Fig 5C and 5D). We then determined the effect of *ddc2-S4* mutation on Mec1 activation in the presence of oligonucleotides (12.5 nM) and RPA (0, 5, 10 nM) (Fig 7E). Mec1-Ddc2-S4, unlike Mec1-Ddc2, was not efficiently stimulated by RPA-ssDNA (Fig 7E). Together, our results support a model in which Ddc2 mediates Mec1 activation through ssDNA recognition while RPA prompts ssDNA binding of Mec1-Ddc2 at sites of DNA damage.

## Discussion

Previous studies have established the model in which ATRIP/Ddc2 interacts with RPA-coated ssDNA and recruits ATR/Mec1 to sites of DNA damage [4, 5]. However, Ddc2 appears to stimulate Mec1 kinase activity at sites of DNA damage [49]. In this study we have further characterized the *ddc2-S4* mutation by carrying out genetic and biochemical experiments. The *ddc2-S4* mutation causes defects in phosphorylation and accumulation of the Rad9 checkpoint mediator at sites of DNA damage. The *ddc2-S4* mutation also confers a defect in phosphorylation of the S-phase specific Mrc1 checkpoint mediator. The Ddc1-Dpb11 axis and Dna2 contribute to Mec1 activation in S phase [26]. Notably, the *ddc2-S4* mutation causes a more significant defect in S phase checkpoint signaling than the *ddc1Δ dna2-AA* mutation. Thus, Ddc2 controls Mec1 activation through a Ddc1/Dpb11/Dna2-independent mechanism. We further examined the effect of *ddc2-S4* mutation on Mec1 kinase activity using an *in vitro* reconstitution system. ssDNA, but not RPA, stimulates Mec1-Ddc2 kinase activity. However, RPA can promote ssDNA-dependent Mec1 activation. Neither ssDNA nor RPA-ssDNA stimulates Mec1-Ddc2-S4 effectively. Our data support a model in which Ddc2 increases Mec1 kinase activity upon ssDNA recognition.

The *ddc2-S4* mutation confers a defect in Rad9 phosphorylation and accumulation at sites of DNA damage. Mec1 phosphorylates Rad9 to allow Rad9-Rad53 interaction and subsequent Rad53 activation [30, 31, 34]. Rad9 accumulates at sites of DNA damage by interacting with K79-methylated histone H3, S129 phosphorylated histone H2A and the scaffold protein Dpb11 [39, 40]. Mec1 phosphorylates Ddc1 to promote Ddc1-Dpb11-Rad9 interaction at sites of DNA damage [39, 44, 48]. In this study we show that the *ddc2-S4* mutation confers a defect in Ddc1 phosphorylation after DNA damage. We have previously shown that histone H2A phosphorylation is decreased in *ddc2-S4* mutants [49]. Thus, two different Rad9 recruitment mechanisms are defective in *ddc2-S4* mutants. Rad9 recruitment defect may compromise Rad9 phosphorylation in *ddc2-S4* mutants because Mec1 accumulates and phosphorylates Rad9 at sites of DNA damage [30, 31, 34]. Mec1 phosphorylates Dpb11 and enhances the stimulating effect of Dbp11 on Mec1 kinase activity *in vitro* [25, 66]. Similar to Dpb11, Ddc1 directly activates Mec1 kinase *in vitro* [21, 23]. It is not known whether phosphorylated Ddc1 stimulates Mec1 kinase activity more effectively than non-phosphorylated one.

The *ddc2-S4* mutation causes a more significant defect in S phase checkpoint activation than the *ddc1Δ dna2-AA* mutation. Notably, the residual checkpoint activation in *ddc1Δ dna2-AA* cells largely depends on Mec1 function. These results suggest that Ddc2 promotes Mec1 activation through a Ddc1/Dbp11/Dna2-independent mechanism. Mec1 phosphorylates the Mrc1 checkpoint mediator and activates Rad53 in S phase [32, 35]. Notably, Mrc1-dependent Rad53 activation does not require Rad9 or Ddc1/Dpb11 function [55]. Recent evidence suggests that the *dna2-AA* mutation affects DNA replication or repair function rather than checkpoint activation [27]. Thus, Ddc2-dependent Mec1 activation appears to play a key role in the stimulation of the Mrc1 checkpoint pathway during S phase.

**Fig 8:**
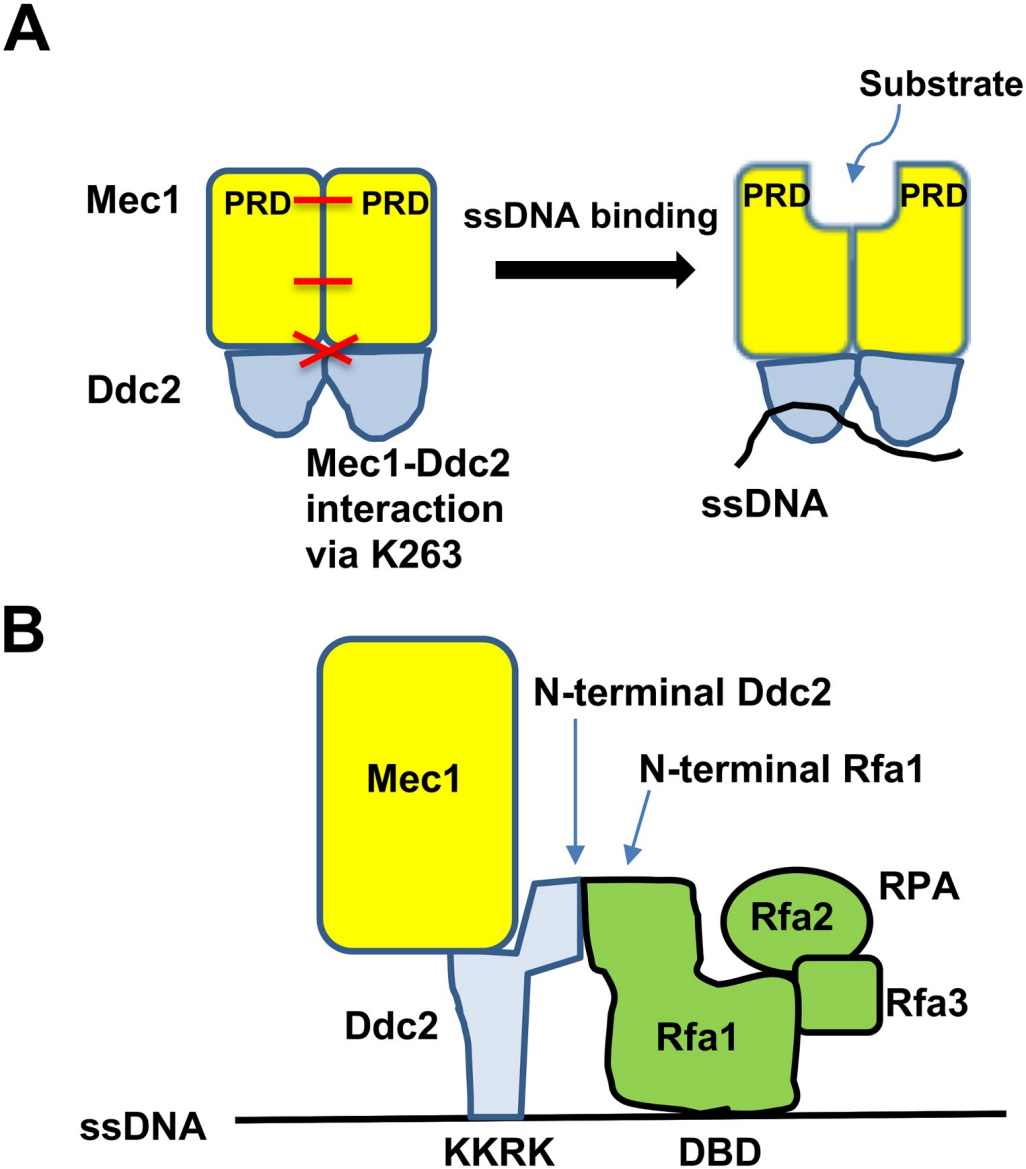
Model for Mec1 activation at RPA-covered ssDNA tracts. (A) ssDNA binding of Ddc2 increases Mec1 activity. ssDNA binding of Ddc2 triggers conformation changes of the entire Mec1-Ddc2 homodimer, resulting in structural changes of the kinase domain at the C-terminus of Mec1. K263 is involved in homodimerization of the Mec1-Ddc2 heterodimer. The PRD-PRD interface within the kinase domain is also involved in homodimerization of the Mec1-Ddc2 heterodimer. See text for more detail. (B) RPA promotes ssDNA-dependent Mec1 activation. The N-terminus of Ddc2 interacts with the N-terminus of Rfa1 whereas the DNA binding (KKRK) region of Ddc2 is involved in ssDNA binding. RPA alone binds to ssDNA through its own DNA binding domain (DBD). See the text for more detail.

The *ddc2-S4* mutation causes a defect in ssDNA-dependent Mec1 activation. Then how does ssDNA stimulate Mec1 kinase activity? Mec1-Ddc2 forms a dimer of heterodimers through multiple interfaces including the PIKK regulatory domain (PRD) [67]. Interestingly, the PRD is closely positioned near the catalytic and activation loop at the kinase domain, thereby blocking kinase activity and substrate entry [61, 67]. We propose that ssDNA binding of Ddc2 triggers conformation changes of the Mec1-Ddc2 homodimer, which could open up the catalytic active site (Fig 8A). The *ddc2-S4* mutation carries two substation mutations (K263E, H382Y). K263 contributes to Mec1-Ddc2 homodimerization [61] (S5 Fig) whereas H382 is positioned near the putative DNA binding (^177^KKRK^180^) region [61] (Fig 5B). Thus, the *ddc2-S4* mutation may affect conformation changes of Mec1-Ddc2 homodimer upon ssDNA binding. Mec1 phosphorylates Mec1 activators (Ddc1, Dpb11 or Dna2) after DNA damage [66, 68, 69]. Conformation changes of the kinase domain could therefore enhance binding of Mec1 activators to its own kinase domain. Consistent with the view, Dpb11 has been shown to activate Mec1 more strongly in the presence of RPA and ssDNA [22].

RPA at lower concentrations promotes ssDNA-dependent Mec1 activation *in vitro*. Ddc2 and RPA recognize ssDNA through different mechanisms; the KKRK motif of Ddc2 is implicated in DNA binding [62] whereas RPA utilizes its own DNA binding domain (DBD) [8] (Fig 8B). However, the N-terminus of Ddc2 interacts with RPA independently of ssDNA although other domains of Ddc2 may be involved in RPA interaction [49, 63, 64] (Fig 8B). Thus, RPA-Ddc2 interaction could stimulate Ddc2-ssDNA binding by providing an additional ssDNA-binding interface, thereby boosting ssDNA-dependent Mec1 activation. However, we cannot fully exclude the possibility that RPA, once coated on ssDNA, acquires the ability to directly stimulate Mec1 activity. RPA at higher concentrations attenuates ssDNA-dependent Mec1 activation *in vitro*. One explanation is that RPA by itself competes with RPA-ssDNA for Mec1-Ddc2 binding. Alternatively, there would be substrate competition between RPA and *in vitro* substrates. At the moment, it remains to be determined which property of RPA down-regulates ssDNA-dependent Mec1 activation *in vitro*. Previous *in vitro* studies have shown that RPA-ssDNA has no apparent impact on ATR-ATRIP/Mec1-Ddc2 kinase activity; however, the effect of different RPA concentrations has not been extensively investigated [21, 22, 63, 70]. RPA not only stabilizes ssDNA but also stimulates various repair processes [8, 71–75]. Moreover, RPA binding to ssDNA is highly dynamic using different binding modes [76, 77]. Interestingly, RPA depletion modulates ssDNA generation and Mec1 activation differently [74]. Thus, dynamic interactions between ssDNA, RPA and Mec1-Ddc2 might be important for efficient Mec1 activation. A high-resolution structure of Mec1-Ddc2 has been recently reported [61]. However, the structure and dynamics of how Mec1-Ddc2 and RPA assemble on ssDNA remain to be elucidated.

In summary, we have shown that Ddc2 promotes Mec1 activation independently of Ddc1, Dpb11 and Dna2. We have also provided evidence supporting that Ddc2 promotes Mec1 activation through ssDNA recognition. ATR/Mec1 recognizes RPA-ssDNA and controls many cellular activities during DNA replication and repair [4, 5]. Our studies thus provide insight into how RPA-containing DNA structures modulate ATR/Mec1 activation, and suggest that ATR/Mec1, like DNA-PK and ATM/Tel1, is activated upon the recruitment to sites of DNA damage.

## Experimental procedures

### Strains and plasmids

pRS424-GAL-FLAG-MEC1 is a high-copy plasmid version of YCp/pRS316-GAL-FLAG-MEC1 [78]. The *GAL1-GAL10* promoter region was amplified by PCR with the primer pair 3016 and 3017, fusing a sequence encoding MEHHHHHH to the *GAL1* promoter. The PCR product was cleaved with EcoRI and MluI. The *DDC2* or *ddc2-S4* coding sequence [49] was amplified by PCR with the primer pair KS460 and KSX001, fusing a HA epitope to the N-terminus of Ddc2 or Ddc2-S4, respectively. The PCR product was cleaved with MluI and SalI. The EcoRI-MluI and the MluI-SalI fragments were cloned into YEplac195, generating YEp195-GAL-His-HA-Ddc2 or YEp195-GAL-His-HA-Ddc2-S4, respectively. The YCpT-Rad53-HA plasmid has been described [49]. The *dna2-W128A*, *Y130A* (*dna2-AA*) mutation [26] was integrated into the own locus after PCR fusion [79] using primers KS2943, KS2944, KS2955 and KS2946. The *MRC1-HA*::*TRP1* construct was generated by a PCR-based method [80] using the primer pair KS3649 and KS3650. The strains used in this study are listed in S1 Table.

### DNA end resection assay

Quantitative PCR analysis of DNA end resection was performed as described previously [51]. HO cleaves the HO cut site and generate a DSB. The DNA was digested with the EcoRI restriction enzyme that cleaves the amplicons at 0.8 kb and 5.8 kb from the DSB, but not in the *SMC2* control region. The ssDNA percentage over total DNA was calculated using the following formula: % ssDNA = [100/[(1+2^**ΔCt^*)/2*]]/*f*, in which ΔCt values are the difference in average cycles between digested template and undigested template of a given time point and *f* is the HO cut efficiency [51]. HO cutting efficiency was determined as described [81]. The oligonucleotides used are listed in S2 Table.

### Protein purification of Mec1-Ddc2 and Mec1-Ddc2-S4

The yeast strain (*mec1*Δ *ddc2*Δ *sml1*Δ; KSC3218) was transformed with pRS424-GAL-FLAG-MEC1 and YEp195-GAL-His-HA-DDC2 or YEp195-GAL-His-HA-DDC2-S4. Transformed cells were grown in sucrose media (2% sucrose 0.05% glucose) to a log-phase and incubated with 2% galactose for 5 hr to induce expression from the *GAL* promoter. Crude extracts were prepared from 10 gram of cells in 50 ml of buffer A (20 mM Tris-HCl [pH 8], 10% glycerol, 3 mM DTT, 0.1% Triton X-100) containing 1 mM EDTA, 100 mM NaCl and inhibitors (1 mM phenylmethylsulfonyl fluoride (PMSF), 1 μg/ml leupeptin, 1 mM benzamidine, 1 mM Na_3_VO_4_) by vortexing with 600 μl of glass beads. After clearing by centrifugation, supernatant was incubated with 2 ml of ANTI-FLAG-M2 affinity agarose (Sigma) for 2 hr. Resin was washed with 20 ml of buffer A containing 400 mM NaCl, 10 ml of buffer A containing 100 mM NaCl, 20 ml of buffer A containing 100 mM NaCl, 5 mM MgCl_2_ and 1mM ATP, and 20 ml of buffer A containing 100 mM NaCl. FLAG-tagged protein was eluted with 4 ml of buffer A containing 100 mM NaCl, 300 μg /ml of FLAG-peptide (Sigma), 2.5 mM MgCl_2_, 5U of Benzonase (Millipore). The FLAG-eluate was incubated with 1 ml of Ni-NTA-agarose (Clontech) for one hour, washed with 5 ml of buffer A containing 100 mM NaCl. Bound protein was eluted with 1.5 ml of buffer A containing 150 mM NaCl and 300 mM imidazole and then concentrated using a Vivaspin 500 column (GE Healthcare) with buffer A containing 150 mM NaCl. All the protein purification procedures were performed at 4°C.

### Purification of MBP-Ddc2 and MBP-Ddc2-S4 protein

The coding sequences for *DDC2* and *ddc2-S4* were amplified by PCR using YCpT-myc-DDC2 [82] and YCp-myc-DDC2-S4 [49] with the primer pair KS3620 and KSX001, and cloned into the BamHI and SalI sites of pMAL-c2X (New England Biolabs) to generate the plasmid pMAL-Ddc2 and pMAL-Ddc2-S4, respectively. Proteins were expressed in *E*. *coli* Rosetta (Novagen) after the incubation with 1 mM IPTG at 30°C for 4 hr. The cell pellet from one liter of culture was suspended in 50 ml of buffer M (25 mM Tris-HCl pH 7.5], 10% glycerol, 0.5 mM EDTA, 1 mM DTT) containing 300 mM NaCl and protease inhibitors (leupeptin and pepstatin A at 5 μg/ml each, 1 mM PMSF). After sonication, crude cell lysates were clarified by centrifugation and then incubated with pre-equilibrated 1 ml of amylose resin (New England Biolabs) for 2 hr. After washing with buffer M containing 1 M NaCl, bound proteins were eluted with 2 ml of buffer M containing 300 mM NaCl and 10 mM maltose. Eluates were pooled and concentrated using Vivaspin 500 columns.

### Binding of Mec1-Ddc2 or MBP-Ddc2 to ssDNA

Streptavidin beads (4 μl; Pierce) coated with biotinylated oligonucleotides were incubated with RPA for 30 min and further incubated for 30 min after the addition of Mec1-Ddc2 or MBP-Ddc2 proteins in 500 μl of the binding buffer B (20 mM Tris-HCl [pH 7.5], 100 mM NaCl, 0.01% NP-40, 10% glycerol, 100 μg/ml bovine serum albumin) at 30°C. Beads were recovered and subjected to immunoblotting analysis.

### Interaction of Mec1-Ddc2 with RPA

Mec1-Ddc2 and RPA were incubated with or without oligonucleotides in the binding buffer B containing ANTI-FLAG-M2 affinity agarose for 30 min at 30°C. Beads were subjected to immunoblotting analysis.

### Mec1 kinase assay

Kinase reactions were carried out by the addition of Mec1-Ddc2 or Mec1-Ddc2-S4 in 40 μl (final volume) of the kinase buffer (20 mM Hepes-KOH [pH 7.5], 10 mM NaCl, 10 mM MgCl_2_, 4 mM MnCl_2_, 50 μM ATP) containing 5 μCi [γ-^32^P] ATP (3,000 Ci/mmol) and 400 nM GST-Rad53. To detect RPA phosphorylation, GST-Rad53 was omitted in kinase reactions. GST-Rad53 was purified as described [49]. Each reaction contains 5 nM purified Mec1-Ddc2 or Mec1-Ddc2-S4. Before initiating kinase reactions, Mec1-Ddc2 or Mec1-Ddc2-S4 was incubated with RPA, ssDNA/oligonucleotide or RPA-ssDNA complex in 4 μl of 15 mM Tris-HCl [pH7.5], 100 mM NaCl, 0.025 mM EDTA for 15 min at 30°C. RPA-ssDNA complex was prepared by mixing RPA and ssDNA/oligonucleotide for 30 min at 30°C. After 10 min of incubation at 30°C, the kinase reactions were terminated by the addition of 5x SDS-sample buffer. The reaction mixtures were separated on SDS-polyacrylamide gels, and phosphorylation was quantified with a phosphor imager system (Typhoon 8600, GE Healthcare).

### Other methods

Cells were incubated with α-factor (6 μg/ml) or nocodazole (15 μg/ml) for 2 hr to synchronize at G1 or G2/M, respectively. Chromatin immunoprecipitation assay and immunoblotting analysis were carried out as described [49, 78]. DNA flow cytometry was carried out by using FACSCalibur (BD Biosciences) [49]. Budding yeast RPA protein was purified as described [83]. Anti-Rfa1 and anti-Rfa2 antibodies were obtained from Steve Brill (Rutgers, Piscataway). Anti-Rad53 antibody (EL7.E1) was purchased from Abcam. The ribbon diagram of Ddc2 was generated by PyMOL (Palo Alto, CA) using the PDB data base (PDB ID: 5X60).

## Supporting information

S1 Fig Overview of the resection assay. DNA end resection generates ssDNA. EcoRI restriction sites become resistant to restriction digestion once converted to ssDNA. PCR amplifies only EcoRI resistant ssDNA.
(TIF)

S2 Fig Purification of Mec1-Ddc2 and Mec1-Ddc2-S4. Purified Mec1-Ddc2 and Mec1-Ddc2-S4 were separated on SDS-PAGE and stained by Coomassie Brilliant Blue. Mec1-Ddc2 (lane 1) and Mec1-Ddc2-S4 (lane 2) were purified through a two-step ANTI-FLAG-M2 and Ni-NTA column purification.
(TIF)

S3 Fig The amount of RPA in purified Mec1-Ddc2 complex. Kinase reactions (40 μl) were carried out with Mec1-Ddc2 (5 nM or 0.2 pmol) using various concentrations (amounts) of RPA in the absence or presence of bio-oligo(dN)_80_ (12.5 nM) as in Fig 7B. Kinase activities of Mec1-Ddc2, normalized to that observed with Mec1-Ddc2 alone, are shown in comparison with those in the presence of RPA or ssDNA. The amount of RPA in purified Mec1-Ddc2 protein was analyzed by immunoblotting analysis with anti-Rfa2 antibody. Purified RPA was loaded as reference.
(TIF)

S4 Fig RPA phosphorylation by Mec1-Ddc2 in the presence or absence of ssDNA. Kinase reactions were carried out using Mec1-Ddc2 (5 nM) with various concentrations of RPA in the absence or presence of bio-oligo(dN)_80_ (125 nM).
(TIF)

S5 Fig Enlarged view of Mec1-Ddc2 homodimerization interface. Ddc2 is shown in green and the Mec1 N-terminal α-solenoid of another Mec1-Ddc2 heterodimer in is highlighted in cyan. The interaction of Lys^263^ of Ddc2 with Asp^322^ and Gln^323^ of Mec1 is shown.
(TIF)

S6 Fig Effect of biotinylated oligonucleotide on Mec1 activation. Kinase reactions were carried out with Mec1-Ddc2 (5 nM) using various concentrations of biotinylated or unmodified oligo(dN) _80_. Incorporation of ^32^P into GST-Rad53 was analyzed as in Fig 6A.
(TIF)

S7 Fig Competition between RPA and ssDNA-bound RPA for Mec1-Ddc2 binding. Because Mec1-Ddc2 interacts with RPA independently of ssDNA, ssDNA-free RPA could compete with ssDNA-bound RPA for Mec1-Ddc2 binding.
(TIF)

S1 Table Strains used in this study. (DOCX)

S2 Table Oligonucleotides in this study. (DOCX)
