# Supplementary figures and images for: Ddc2ATRIP promotes Mec1ATR activation at RPA-ssDNA tracts
Source: PLoS Genet. 2019 Aug 1;15(8):e1008294. doi: 10.1371/journal.pgen.1008294 (PMC6692047; doi:10.1371/journal.pgen.1008294)

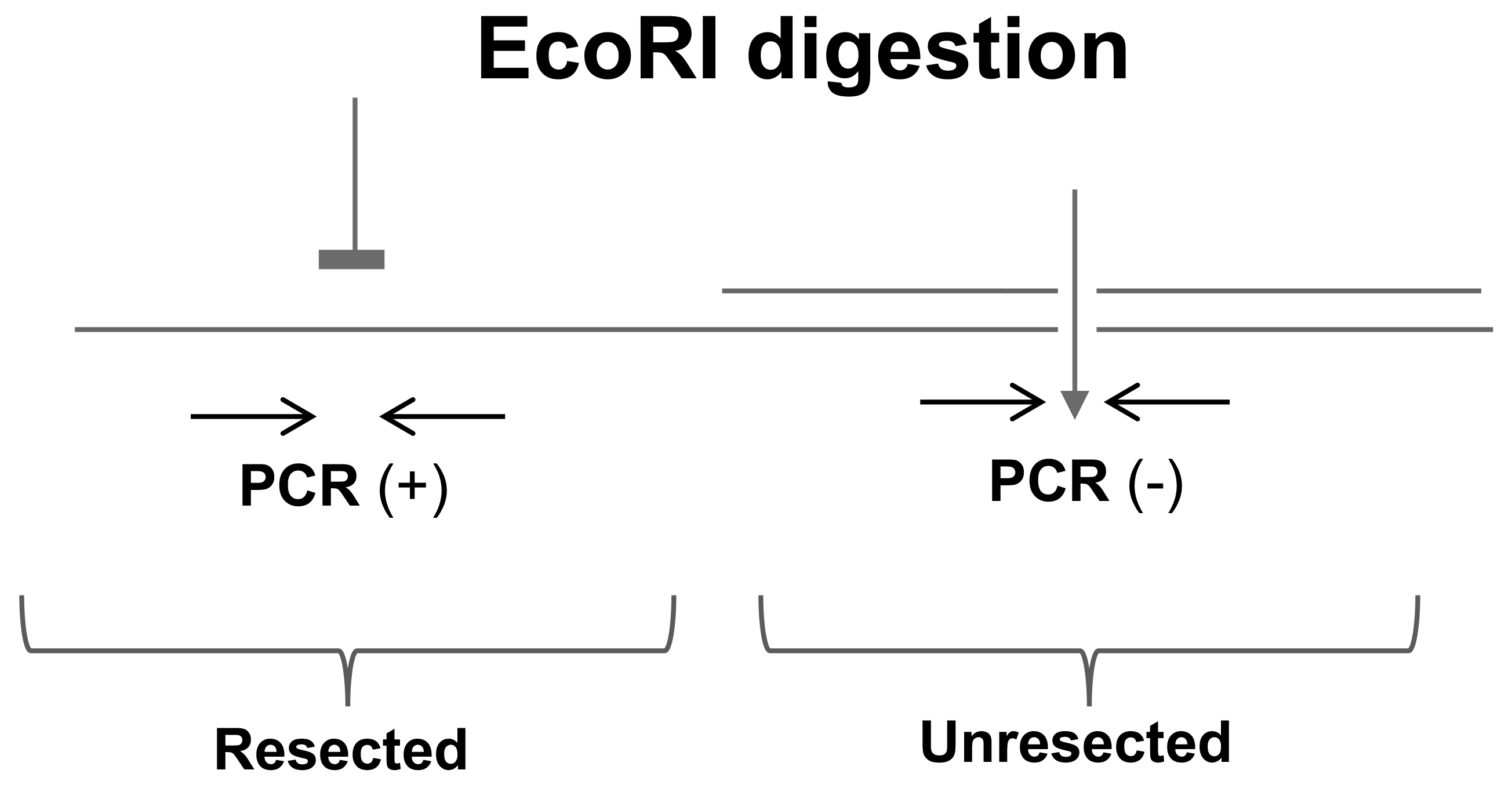

Supplement: S1 Fig — DNA end resection generates ssDNA. EcoRI restriction sites become resistant to restriction digestion once converted to ssDNA. PCR amplifies only EcoRI resistant ssDNA. (TIF) [file pgen.1008294.s001.tif]

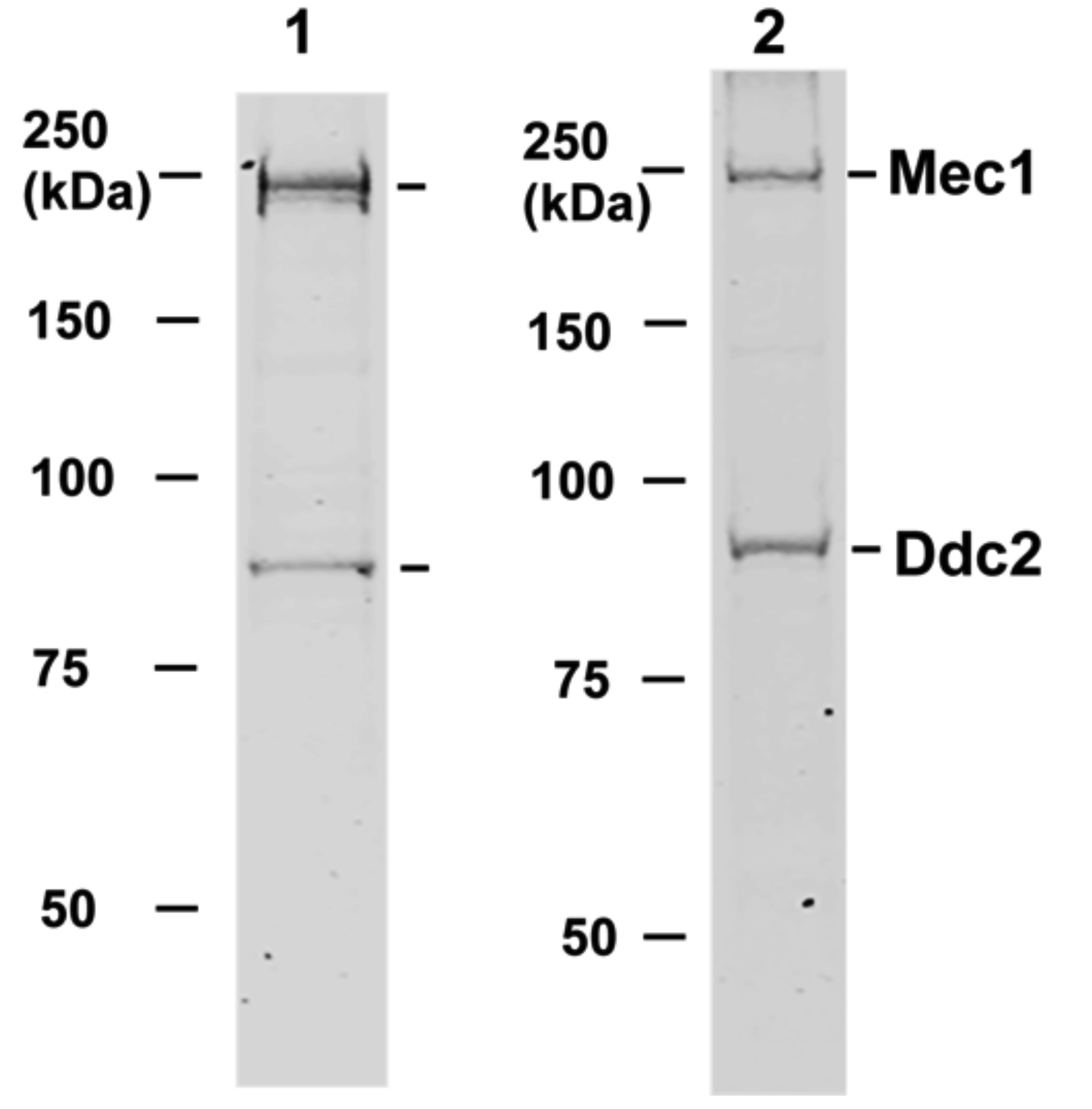

Supplement: S2 Fig — Purified Mec1-Ddc2 and Mec1-Ddc2-S4 were separated on SDS-PAGE and stained by Coomassie Brilliant Blue. Mec1-Ddc2 (lane 1) and Mec1-Ddc2-S4 (lane 2) were purified through a two-step ANTI-FLAG-M2 and Ni-NTA column purification. (TIF) [file pgen.1008294.s002.tif]

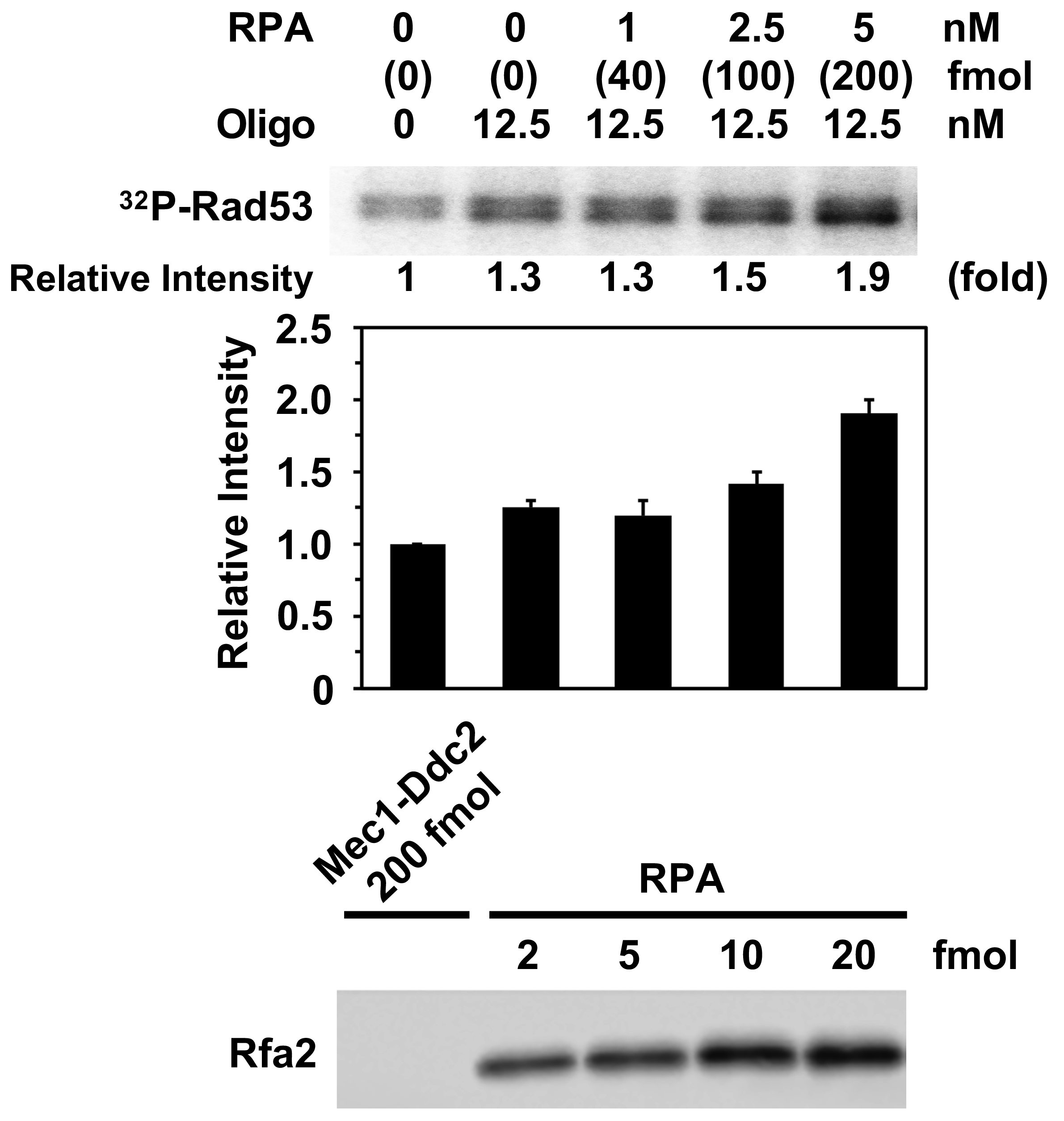

Supplement: S3 Fig — Kinase reactions (40 μl) were carried out with Mec1-Ddc2 (5 nM or 0.2 pmol) using various concentrations (amounts) of RPA in the absence or presence of bio-oligo(dN)80 (12.5 nM) as in Fig 7B. Kinase activities of Mec1-Ddc2, normalized to that observed with Mec1-Ddc2 alone, are shown in comparison with those in the presence of RPA or ssDNA. The amount of RPA in purified Mec1-Ddc2 protein was analyzed by immunoblotting analysis with anti-Rfa2 antibody. Purified RPA was loaded as reference. (TIF) [file pgen.1008294.s003.tif]

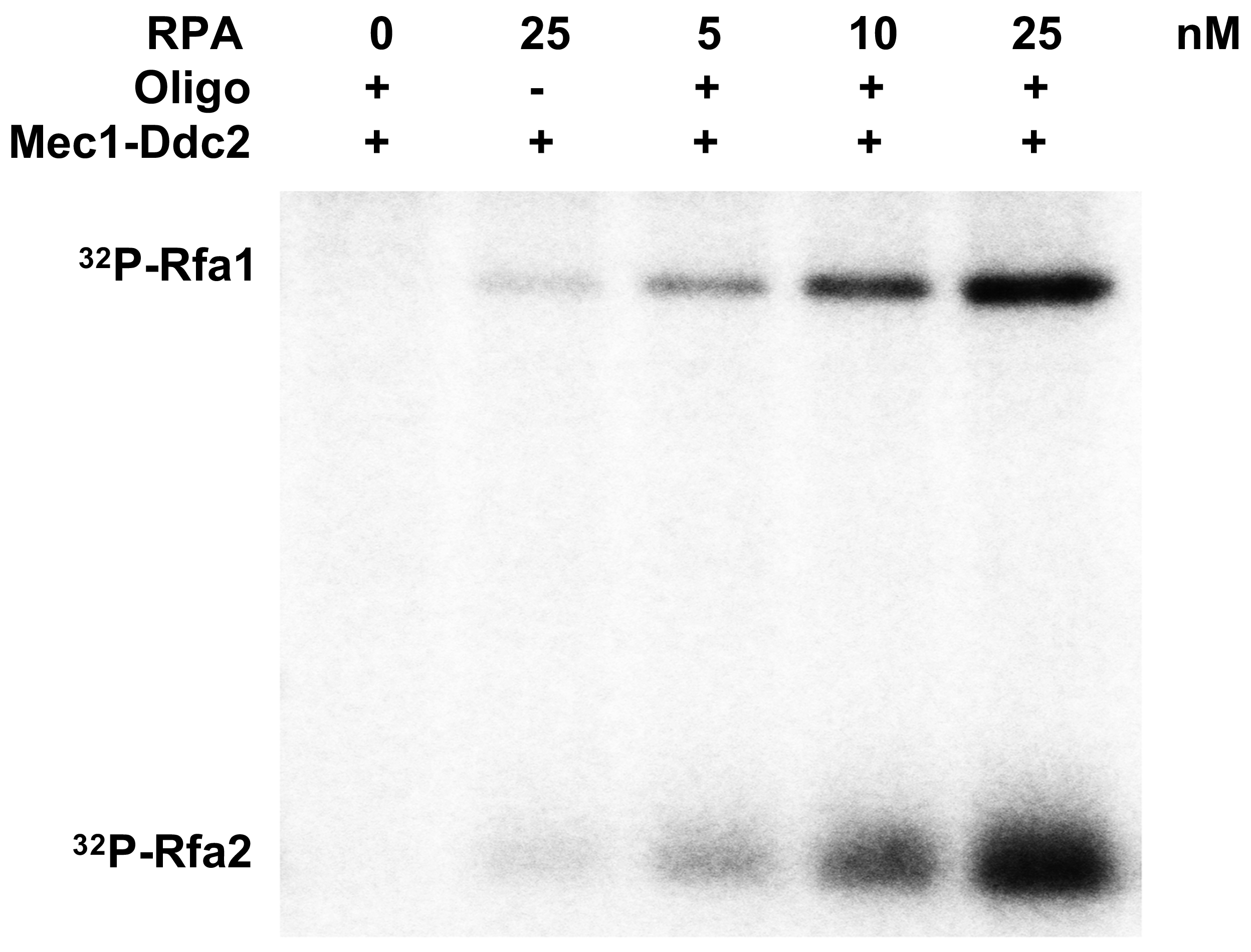

Supplement: S4 Fig — Kinase reactions were carried out using Mec1-Ddc2 (5 nM) with various concentrations of RPA in the absence or presence of bio-oligo(dN)80 (125 nM). (TIF) [file pgen.1008294.s004.tif]

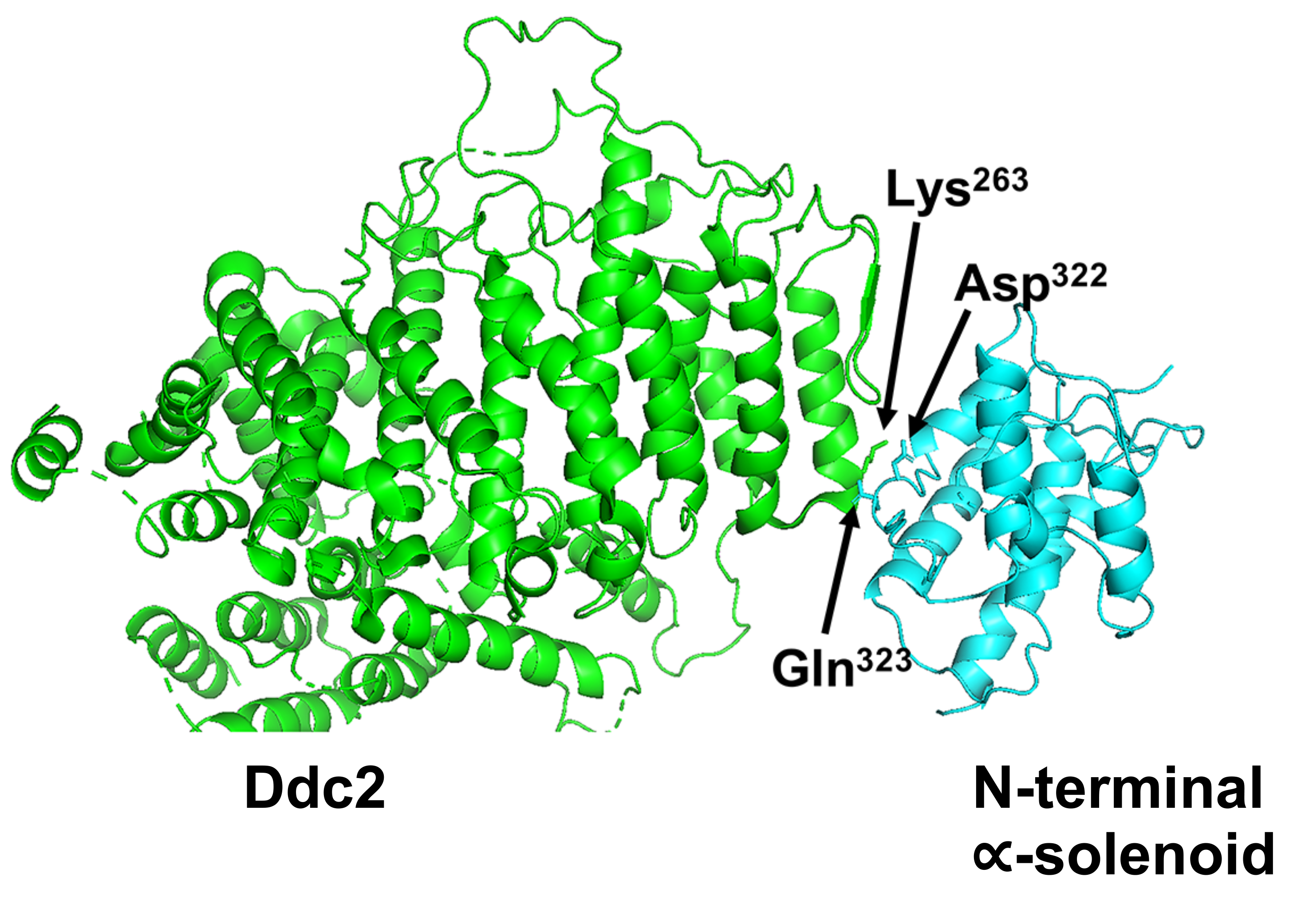

Supplement: S5 Fig — Ddc2 is shown in green and the Mec1 N-terminal α-solenoid of another Mec1-Ddc2 heterodimer in is highlighted in cyan. The interaction of Lys263 of Ddc2 with Asp322 and Gln323 of Mec1 is shown. (TIF) [file pgen.1008294.s005.tif]

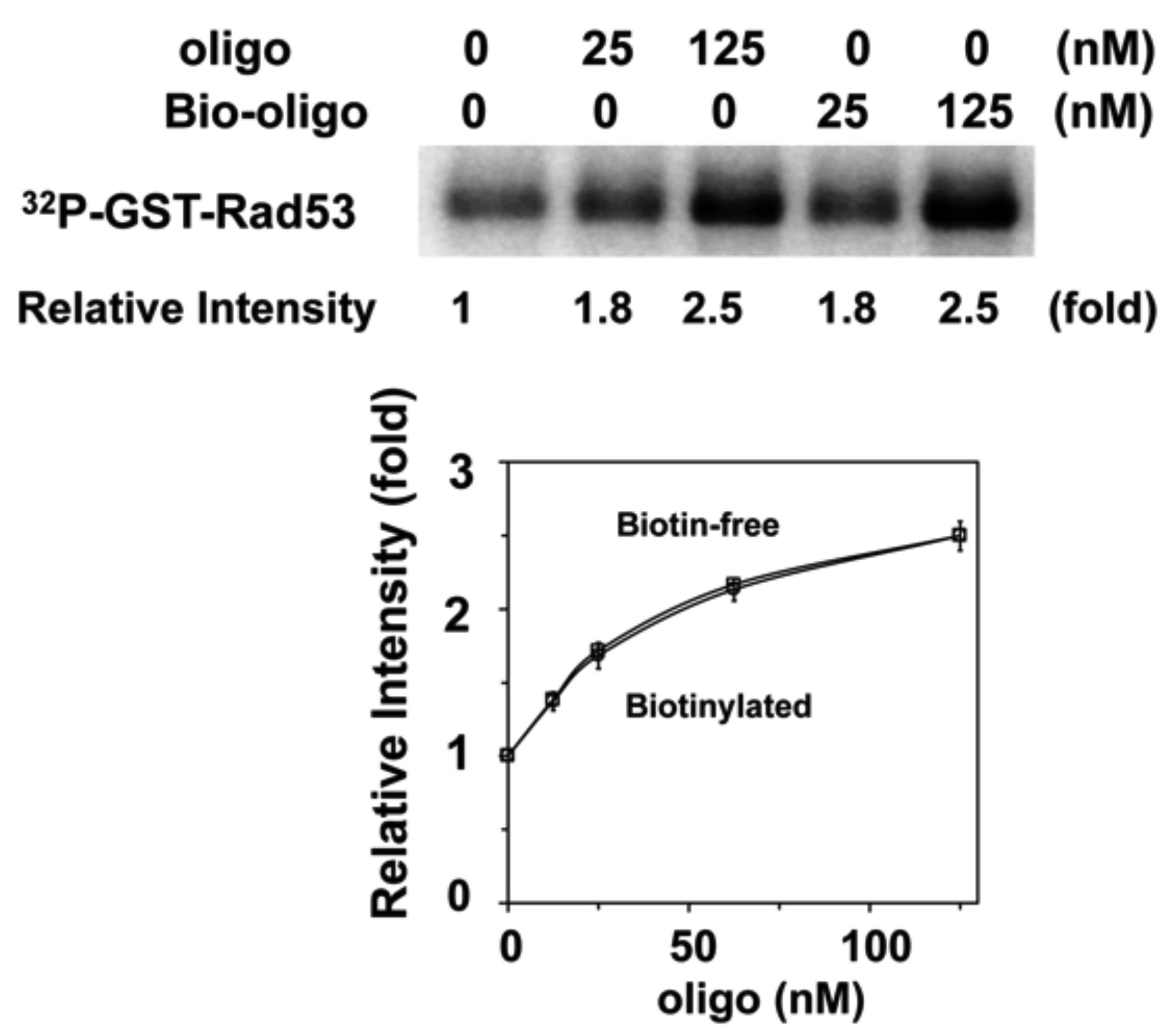

Supplement: S6 Fig — Kinase reactions were carried out with Mec1-Ddc2 (5 nM) using various concentrations of biotinylated or unmodified oligo(dN) 80. Incorporation of 32P into GST-Rad53 was analyzed as in Fig 6A. (TIF) [file pgen.1008294.s006.tif]

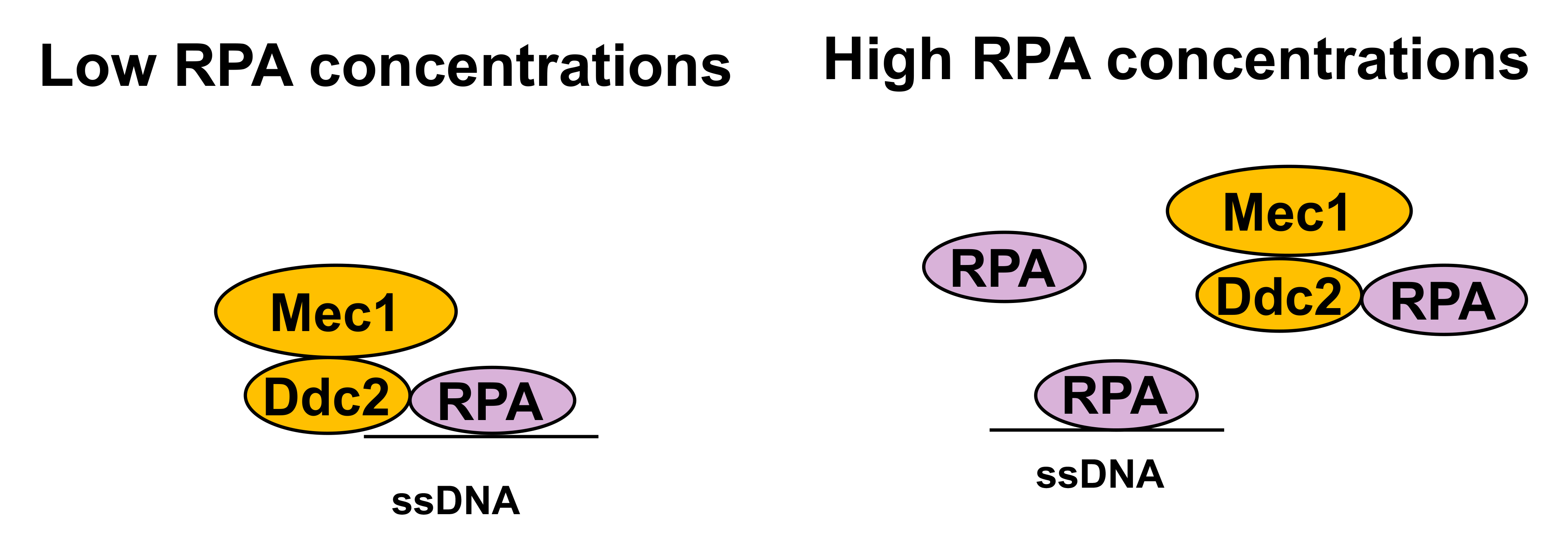

Supplement: S7 Fig — Because Mec1-Ddc2 interacts with RPA independently of ssDNA, ssDNA-free RPA could compete with ssDNA-bound RPA for Mec1-Ddc2 binding. (TIF) [file pgen.1008294.s007.tif]
