# Supplementary material for: Ddc2ATRIP promotes Mec1ATR activation at RPA-ssDNA tracts
Source: PLoS Genet. 2019 Aug 1;15(8):e1008294. doi: 10.1371/journal.pgen.1008294 (PMC6692047; doi:10.1371/journal.pgen.1008294)
Supplement: S1 Table — (DOCX) [file pgen.1008294.s008.docx]

**Table S1. Strains used in this study**

_________________________________________________________________________________________________

Strain Genotype

____________________________________________________________________________________________________________

HB01  *MAT***a***-inc sml1∆::HphMX ADH4cs::HIS2*

HB02 *MAT***a***-inc ddc2-S4::URA3 sml1∆::HphMX ADH4cs::HIS2*

HB09 *MAT***a***-inc RAD9-HA::LEU2 sml1∆::HphMX ADH4cs::HIS2*

HB10 *MAT***a***-inc RAD9-HA::LEU2 ddc2-S4::URA3 sml1∆::HphMX ADH4cs::HIS2*

HB12  *MAT***a***-inc DDC1-HA::LEU2 sml1∆::HphMX ADH4cs::HIS2*

HB13  *MAT***a***-inc DDC1-HA::LEU2 ddc2-S4::URA3 sml1∆::HphMX ADH4cs::HIS2*

KSC4233 *MAT***a** *MRC1-HA::TRP1* *sml1∆::LEU2 ADH4cs::HIS2*

KSC4234 *MAT***a** *MRC1-HA::TRP1 ddc2-S4::URA sml1∆::LEU2 ADH4cs::HIS2*

KSC4235 *MAT***a** *MRC1-HA::TRP1 ddc2∆::LEU2 sml1∆::LEU2 ADH4cs::HIS2*

KSC1178 *MAT***a** *sml1∆::LEU2*

KSC4219 *MAT***a** *ddc1∆::LEU2 dna2-AA::URA3* *sml1∆::HphMX*

KSC3153 *MAT***a** *ddc2-S4::URA3 sml1∆::LEU2*

KSC1234 *MAT***a** *ddc2∆::LEU2 sml1∆::LEU2*

KSC1186 *MAT***a** *mec1∆::LEU2 sml1∆::LEU2*

KSC3218 *MAT***a** *mec1∆::LEU2 ddc2∆::LEU2 sml1∆::LEU2*

__________________________________________________________________________________________________

All the strains are isogenic to KS006 (*MAT***a,** *ade1*, *his2*, *leu2*, *trp1*, *ura3*).
