# Supplementary material for: Ddc2ATRIP promotes Mec1ATR activation at RPA-ssDNA tracts
Source: PLoS Genet. 2019 Aug 1;15(8):e1008294. doi: 10.1371/journal.pgen.1008294 (PMC6692047; doi:10.1371/journal.pgen.1008294)
Supplement: S2 Table — (DOCX) [file pgen.1008294.s009.docx]

**Table S2. List of oligonucleotides**

**Oligonucleotides used for plasmid construction and genetic studies**

**KSX001** AGCGGATAACAATTTCACACAGGA

**KS460** CTCACGCGTATCCATACGATGTTCCAGATTACGCGGAAACGGTGGGTGAATTTTCTTC

**KS823 (HO)** GCTACCTTGAGGAAAGCCAATCACC

**KS824 (HO)** GACATTGCATGTTGGCCTCTTGAAC

**KS827 (0.8 kb)** TCTATTAATGAGCCGAGACCGGTA

**KS828 (0.8 kb)** CGCATGTGAATGACACACGAAAGT

**KS1243 (5.8 kb)** GAATGACAATGAGAATGGTACTGC

**KS1244 (5.8 kb)** TGAAGGACAATTCTCTTAGATCCT

**KS2037 (SMC2)** AAAGACTGAGCTGAATGAAGTCTC

**KS2038 (SMC2)** CGTCTGAGAATCTTGAACAACAAC

**KS2943** GTCGACCTGCAGCGTACGGAACTACTTCAAAGCTACGC

**KS2943** ACGGAGAGACTCCCTGGTAG

**KS2943** CGAGCTCGAATTCATCGATGTGTGGTAGGTAGCAACTAC

**KS2943** ATGGCTGCGTCTGAAGTAGC

**KS3649**  ACAAAATCGAATAAACTTTTTGAAAGCGGACAAGATAGCTTTGATAATCGTACGCTGCAGGTCGAC

**KS3650**  AATCCTATCATAACATGACTATGGCTTGGCCTAGACTCGGGTGCCATCTAATACGAC

TCACTATAGGG

**Oligonucleotides used for biochemical assays**

**Oligo(dN)_80_**

CTCTGAATTCACACCCACACACCACCACACCCACACACCAAGCGGATAACAATTTCACACCTCTTCTCTTCTCTTCTCTT

**Bio-oligo(dN)_80_**

biotin-CTCTGAATTCACACCCACACACCACCACACCCACACACCAAGCGGATAACAATTTCACACCTCTTCTCTTCTCTTCTCTT

**Oligo(dT)_80_**

TTTTTTTTTTTTTTTTTTTTTTTTTTTTTTTTTTTTTTTTTTTTTTTTTTTTTTTTTTTT TTTTTTTTTTTTTTTTTTTT

**Oligo(dT)_60_**

TTTTTTTTTTTTTTTTTTTTTTTTTTTTTTTTTTTTTTTTTTTTTTTTTTTTTTTTTTTT

**Oligo(dT)_40_**

TTTTTTTTTTTTTTTTTTTTTTTTTTTTTTTTTTTTTTTT

**Oligo(dT)_20_**

TTTTTTTTTTTTTTTTTTTT
